# Isolation, Characterization, and Antibacterial Activity of Hard-to-Culture Actinobacteria from Cave Moonmilk Deposits

**DOI:** 10.3390/antibiotics7020028

**Published:** 2018-03-22

**Authors:** Delphine Adam, Marta Maciejewska, Aymeric Naômé, Loïc Martinet, Wouter Coppieters, Latifa Karim, Denis Baurain, Sébastien Rigali

**Affiliations:** 1 Integrative Biological Sciences (InBioS), Center for Protein Engineering, Liège University, B-4000 Liège, Belgium; delphine.adam@doct.uliege.be (D.A.); maciejewska.m@wp.pl (M.M.); Aymeric.Naome@uliege.be (A.N.); lmartinet@doct.uliege.be (L.M.); 2 Genomics Platform, GIGA (Grappe Interdisciplinaire de Génoprotéomique Appliquée), University of Liège (B34), B-4000 Liège, Belgium; wouter.coppieters@ulg.ac.be (W.C.); lkarim@ulg.ac.be (L.K.); 3 Integrative Biological Sciences (InBioS), PhytoSYSTEMS, Eukaryotic Phylogenomics, University of Liège, B-4000 Liège, Belgium; Denis.Baurain@uliege.be

**Keywords:** Cave microbiology, secondary metabolite, antibiotics, rare Actinobacteria, Streptomyces, Amycolatopsis, unculturability, siderophore

## Abstract

Cave moonmilk deposits host an abundant and diverse actinobacterial population that has a great potential for producing novel natural bioactive compounds. In our previous attempt to isolate culturable moonmilk-dwelling Actinobacteria, only *Streptomyces* species were recovered, whereas a metagenetic study of the same deposits revealed a complex actinobacterial community including 46 actinobacterial genera in addition to streptomycetes. In this work, we applied the rehydration-centrifugation method to lessen the occurrence of filamentous species and tested a series of strategies to achieve the isolation of hard-to-culture and rare Actinobacteria from the moonmilk deposits of the cave “Grotte des Collemboles”. From the “tips and tricks” that were tested, separate autoclaving of the components of the International Streptomyces Project (ISP) medium number 5 (ISP5) medium, prolonged incubation time, and dilution of the moonmilk suspension were found to most effectively improve colony forming units. Taxonomic analyses of the 40 isolates revealed new representatives of the *Agromyces*, *Amycolatopsis*, *Kocuria*, *Micrococcus*, *Micromonospora*, *Nocardia*, and *Rhodococcus* species, as well as additional new streptomycetes. The applied methodologies allowed the isolation of strains associated with both the least and most abundant moonmilk-dwelling actinobacterial operational taxonomic units. Finally, bioactivity screenings revealed that some isolates displayed high antibacterial activities, and genome mining uncovered a strong potential for the production of natural compounds.

## 1. Introduction 

Bioprospecting for natural compounds from microorganisms dwelling in poorly explored and extreme environments has gained renewed interest [1,2], boosted by the need to fight resistance to compounds currently used as antimicrobials, herbicides, antivirals, and anticancer agents [3]. Caves, despite being highly oligotrophic environmental niches, support rich and diverse microbial life, a phenomenon called “the Paradox of the Plankton”, which suggests that, in spite of limited nutrient resources, an unexpectedly wide range of species coexist [4]. The extremely starved nature of subsurface habitats presumably stimulates unique strategies of indigenous microbiomes, among which the fine-tuning of the secondary metabolism (in terms of quantity and diversity of molecules) might be one of the key features enabling life in such challenging environments [5,6]. The observation that Actinobacteria, which are the most prolific producers of specialized (secondary) metabolites [7], are also abundant in limestone caves [8,9,10] is not likely a coincidence. Therefore, Actinobacteria isolated from oligotrophic environments, particularly the rare representatives of the phylum, are expected to own a unique metabolome that could potentially be an important source of novel drugs.

In our first attempt, aimed at isolating antibiotic-producing Actinobacteria from cave carbonate deposits called moonmilk originating from the cave “Grotte des Collemboles”, we successfully isolated representatives of a single genus, *Streptomyces* [11,12]. However, high-throughput sequencing (HTS) of DNA extracted from the same moonmilk deposits demonstrated that we failed to isolate representatives of at least 46 additional actinobacterial genera [13]. Such an outcome might be related not only to the fact that *Streptomyces* are adapted to use a wide range of nutrients and therefore grow effectively on many selective media, but also to the fact that their growth is faster in comparison to that of other actinobacterial genera. For this reason, non-streptomycetes, which are more challenging to isolate in pure cultures, are regarded as rare Actinobacteria. Nonetheless, the 78 culturable *Streptomyces* strains isolated in our previous screening [11] constitute a minor fraction of those dwelling in the studied moonmilk deposits, because we only recovered relatives of 5 out of the 19 operational taxonomic units (OTUs) affiliated with the *Streptomyces* genus [13], suggesting that this environment hosts hard-to-culture *Streptomyces* species that are potentially highly diverged from their soil-dwelling counterparts. 

Bacterial unculturability is one of the major problems in microbiology, as many species are present in the environment in a so-called viable but non-cultivable state (VBNC) [14]. The phenomenon known as the “great plate count anomaly” clearly depicts this issue—no matter what kind of approach will be applied to the isolation of microorganisms, it is possible to cultivate only about 1% of what is present in a sample [15]. As demonstrated in the recently published new “Tree of Life”, the large majority of “known” microorganisms have been identified exclusively via genome-based approaches and are not represented by any culturable strain [16]. For this reason, a series of innovations in cultivation techniques are being introduced to increase the recovery yield of microorganisms from their natural habitats, including: (i) dilution of the sample to minimize the competition of fast-growing organisms and reduce the effect of growth inhibitors [17]; (ii) addition of signaling compounds/growth factors [18,19]; (iii) extension of the incubation time for the recovery of slow-growing bacteria [20]; (iv) in situ cultivation [21]; (v) use of polymers as growth substrates to reduce osmotic shock [20]; and (vi) limitation of the production of reactive oxygen species, which prevent bacterial growth [22,23]. In addition, various pretreatment methods are being applied to selective isolation, which targets (to favor or instead to exclude) a bacterial group of interest [24]. In the case of Actinobacteria, a series of chemical and physical treatments are used [20], which take advantage of the high resistance of actinobacterial spores to many factors such as ultraviolet (UV), ultrasonic waves, desiccation, and many chemicals [25,26,27].

In light of the above findings, and taking into account the fact that moonmilk deposits host a broad diversity of Actinobacteria (243 OTUs) [13], we have made an attempt to improve their recovery, particularly of rare taxa, in pure cultures by combining several approaches. Here, we present the “tips and tricks” used to isolate rare moonmilk-dwelling Actinobacteria, evaluate their potential at producing antibacterial compounds, and describe their genetic capacity to produce other specialized metabolites.

## 2. Results and Discussion

### 2.1. Assessment of Various Strategies for Isolating Moonmilk-Dwelling Rare Actinobacteria

Different strategies were implemented in order to increase the odds of isolating hard-to-cultivate Actinobacteria from the moonmilk samples of the cave “Grotte des Collemboles”. For this purpose, we applied the rehydration-centrifugation (RC) method to the preparation of the moonmilk suspension. Applying the RC methodology has been shown previously to lessen the occurrence of filamentous and long spore-chain forming species (such as most *Streptomyces*) and non-motile bacteria, thereby enriching the isolation of rare and zoosporic Actinobacteria [27]. The moonmilk suspension was inoculated in the two media that showed the most contrasting isolation efficiency in our previous screening [12]—(i) the International Streptomyces Project (ISP) medium number 5 (ISP5) medium in which no colonies could be observed; and (ii) the starch nitrate (SN) medium, which gave the highest number of colony forming units (CFUs). As the existence of unculturable bacteria in certain environments has been proposed to depend on siderophore-producing neighboring microorganisms [18,28], we facilitated iron acquisition in these two media by (i) increasing the concentration of iron (20 µM FeCl_3_); (ii) including 1 µM of the siderophore desferrioxamine B (DFB), which has been shown to be essential for growth in certain media [28]; and (iii) adding both FeCl_3_ and DFB. Moreover, in order to reduce the formation of highly toxic reactive oxygen species (ROS) that might be generated during autoclaving through the interaction of components of the media, particularly phosphate and agar [22,23], ISP5 and SN were prepared in two different manners—(i) either with all of the components autoclaved together or (ii) with agar and phosphate autoclaved separately from all of the other components. Further evaluation of the possible toxic effect of ROS generated during autoclaving was tested by adding a solution of catalase to the center of the plates, as described previously [23]. All media were additionally supplemented with selective agents (antibiotic and antifungal compounds), inoculated with serially diluted moonmilk suspension (up to 10^−4^), and incubated for up to two months.

**Figure 1:**
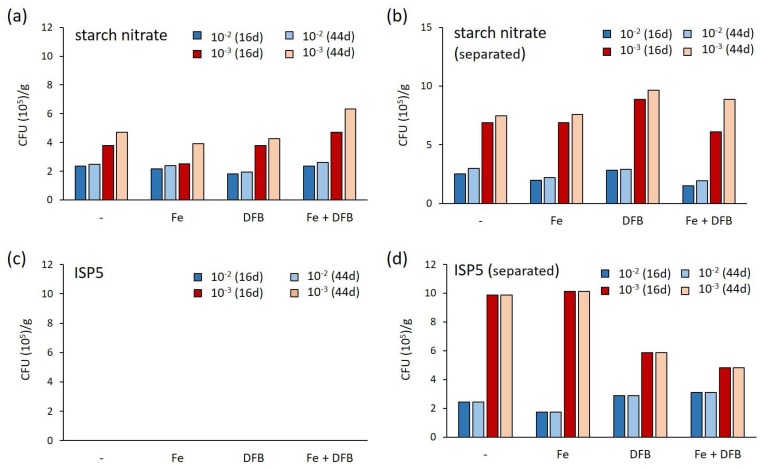
Total number of colony forming units (CFUs) according to the different media and methods used. The different media were inoculated with a diluted moonmilk suspension (10^−2^ in blue and 10^−3^ in red) prepared according to the rehydration-centrifugation method and incubated for up to two months. Data obtained after 16 days (16 d) and 44 days (44 d) of incubation are displayed. The “separated” label indicates that the autoclaving of agar and phosphate was performed separately from the other components of the media. Abbreviations: Fe, 20 μM of FeCl_3_; DFB, 1 μM of desferrioxamine B.

The combination of strategies described above increased the magnitude of isolated bacteria by about 15-fold in comparison to the colony counts obtained in our previous screening (~5 × 10^5^ CFUs per gram of inoculum in this study (Figure 1), in contrast to ~3 × 10^4^ CFUs in [12]). Dilution of the inoculum (from 100- to 1000-fold) increased CFUs (compare bar plots in blue and those in red in Figure 1). Considering that bacteria living in extremely oligotrophic environments are thought to have developed mutualistic interactions, this result is rather unexpected. Indeed, dilution increases the distance between colonies—which limits the exchange of growth factors between ‘helpers’ and unculturable organisms—and should eventually yield a lower number of colonies. The fact that diluting the inoculum instead resulted in higher CFUs suggests either that one or more of the strains inoculated at the lower dilution secreted highly active antibacterial agents or that the collected moonmilk deposits could possibly contain growth inhibitors that were attenuated by the sample dilution.

**Figure 2:**
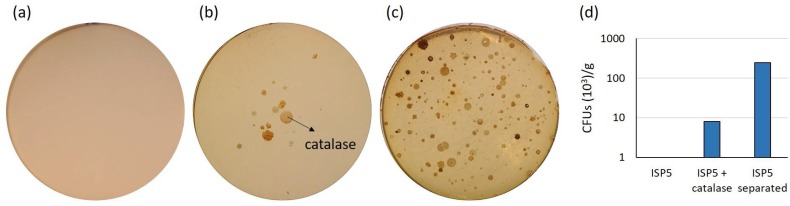
Effect of reactive oxygen species on bacterial growth in International Streptomyces Project (ISP) medium number 5 (ISP5) medium. Growth in (**a**) ISP5 medium prepared according to the standard protocol, in (**b**) the same medium but with catalase injected on the paper disc in the center of the plate, and in (**c**) the ISP5 medium prepared with autoclaving of agar and phosphate separate from the other media components. (**d**) CFUs on (**a**), (**b**) and (**c**).

Furthermore, the putative effect of ROS on bacterial cultivability was clearly recorded on the ISP5 medium (Figure 1 and Figure 2). Sterilization of phosphate and agar separate from the other components of the ISP5 medium resulted in abundant growth, whereas no colonies could be observed when the same medium was prepared according to the standard procedure (Figure 1 and Figure 2). The probable effect of ROS-preventing bacterial growth on the traditionally prepared ISP5 medium was supported by the observation that adding a solution containing a catalase (decomposing hydrogen peroxide to water and oxygen) restored colony formation (Figure 2b). In contrast, supplementation of the media by iron (at final concentration of 20 μM), DFOB (at a final concentration of 1 μM), or both did not significantly improve the bacterial count (Figure 1). However, regardless of the lack of improved CFUs, the growing/isolated bacteria still might be different from those grown in the same media deprived from additional iron/siderophore supply. Finally, increased incubation time (from 16 to 44 days) increased CFUs in only a limited number of conditions (see for instance Fe + DFB in starch nitrate medium, Figure 1).

### 2.2. Characterization of Culturable Moonmilk-Derived Actinobacterial Isolates 

100 colonies were initially selected for further characterization, but only 42 survived after two rounds of inoculation/cultivation to obtain pure isolates (Table 1). This significant loss (58%) was likely caused by the lack of growth factors emanating from neighboring colonies in pure cultures. Hence, the strains that passed the purification steps might represent bacteria able to feed only on nutrients present in the synthetic medium, whereas species most adapted to cooperative growth and life in oligotrophic and inorganic environments would constitute a significant part of the lost isolates. The remaining 42 isolates were cultured in liquid LB, ISP1, or ISP2 media for DNA isolation and subsequent sequencing of the 16S (SSU) rRNA gene, either at full length (extracted from the genome sequence) or nearly full length (through PCR amplification).

**Figure 3:**
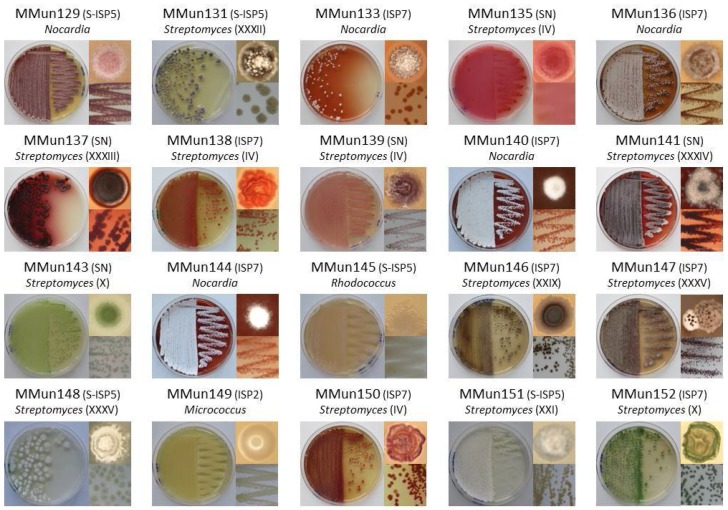

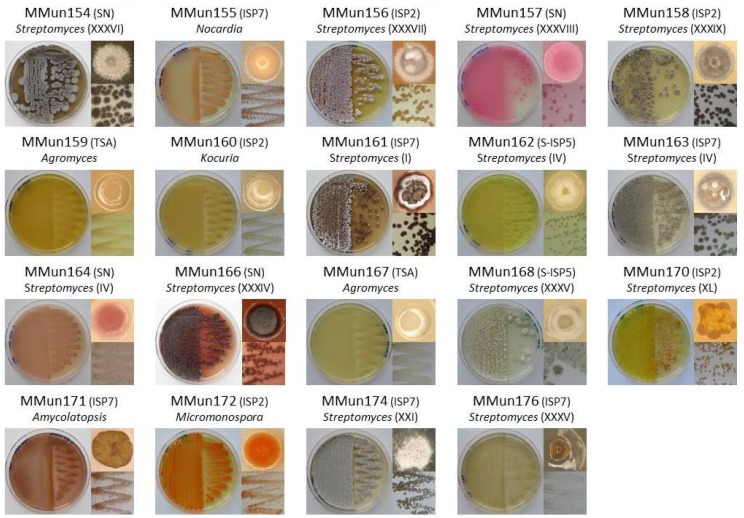
Phenotypes of actinobacterial strains isolated in this study. For each isolate, the front and back of the Petri dish-grown bacteria are presented together with the phenotype of a single colony. The phylotype number for *Streptomyces* isolates is indicated in parentheses.

Table 1 lists the closest hits for members of our collection deduced from BLAST searches, and Figure 3 shows their phenotype. Two of the isolates (MMun130 and MMun142), closely related to *Paenibacillus* sp. DSL09-3 and *Paracoccus* sp. clone 54, respectively (Table 1), are not Actinobacteria and were not investigated further in this study. The remaining 40 isolates represented eight actinobacterial genera, namely *Agromyces*, *Amycolatopsis*, *Kocuria*, *Micrococcus*, *Micromonospora*, *Nocardia*, *Streptomyces*, and *Rhodococcus* (Table 1 and Figure 3). Finding members of the *Rhodococcus* genus, which was the most abundant in terms of the absolute number of sequences (17% of actinobacterial 16S rRNA sequences), and of the *Streptomyces* genus, which displayed the largest diversity (19 OTUs) based on DNA extracted from the investigated moonmilk deposits [13], was rather expected. However, finding representatives of genera *Nocardia* (0.5%), *Agromyces* (0.2%), *Kocuria* (0.1%), and *Amycolatopsis* (0.03%), which correspond to less abundantly represented taxa, demonstrated the efficiency of the tested cultivation approaches to also recover rare moonmilk-dwelling Actinobacteria. Additionally, our work reports the first isolation of strains belonging to the *Micrococcus*, *Kocuria* (Micrococcaceae), and *Micromonospora* (Micromonosporaceae) genera from moonmilk deposits. While other members of Micrococcaceae (*Arthrobacter*) had been previously reported in moonmilk [10], representatives of the Micromonosporaceae family (MMun172) had not been identified so far in this type of speleothems.

**Figure 4:**
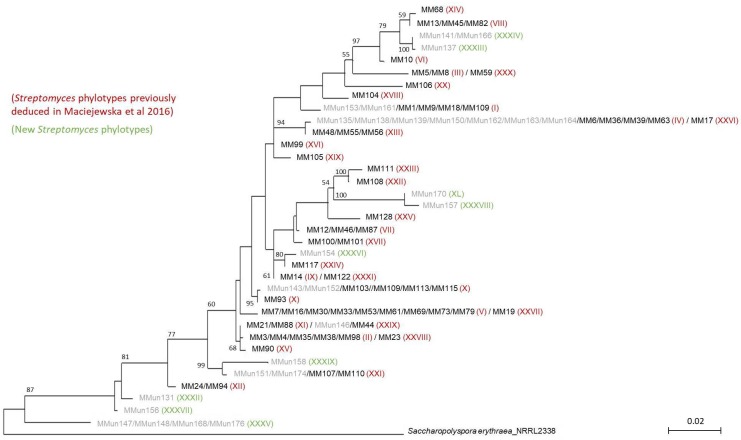
16S rRNA-based phylogenetic tree of moonmilk-dwelling *Streptomyces* strains. Strains isolated using the rehydration-centrifugation (RC) method (MMun) are shown in grey, and strains isolated in our previous study (MM) are in black. Phylotype affiliations are indicated in parentheses. The alignment of nearly complete 16S rRNA sequences had 1538 unambiguously aligned positions for 92 strains, but only the 1342 positions without missing nucleotides were used to infer the tree. The evolutionary model was GTR + Γ_4_ and bootstrap values are based on 100 pseudoreplicates (bootstrap values <50% are not displayed). *Saccharopolyspora erythraea* was used as outgroup. The scale bar represents 0.02 substitutions per site.

The overrepresentation of *Streptomyces* isolates (27 out of 40 strains) despite using the RC protocol might be explained by specific morphological features of the isolated moonmilk-dwelling species. While *Streptomyces* filamentous pellets would be excluded from the final suspension by the centrifugation steps in the RC protocol, the inoculum could still contain single streptomycetes spores. *Streptomyces lunaelactis* [29], the most frequently observed *Streptomyces* species in the studied deposits, was found to form collapsed/fragmented packs of spores instead of long aerial spore chains. This morphological feature would explain why we isolated two strains of the *S. lunaelactis* species (MMun143 and MMun152) using the RC method and could be a common characteristic of the other *Streptomyces* isolates obtained in this study. In addition to these two novel *S. lunaelactis* strains, another 14 out of 27 *Streptomyces* strains obtained in this study have an identical 16S rRNA sequence compared to strains previously isolated from the same moonmilk deposits (MM18, MM44, MM63, MM82, MM110, MM126 [12], Figure 4 and Table 1). These 14 “MMun” strains were therefore assigned to phylotypes defined in our first screening (phylotypes marked with an asterisk in Table 1) [12], and the other 11 *Streptomyces* strains were distributed into 9 novel phylotypes (from XXXII to XL) based on their 16S rRNA sequence (Figure 4). 

**Figure 5:**
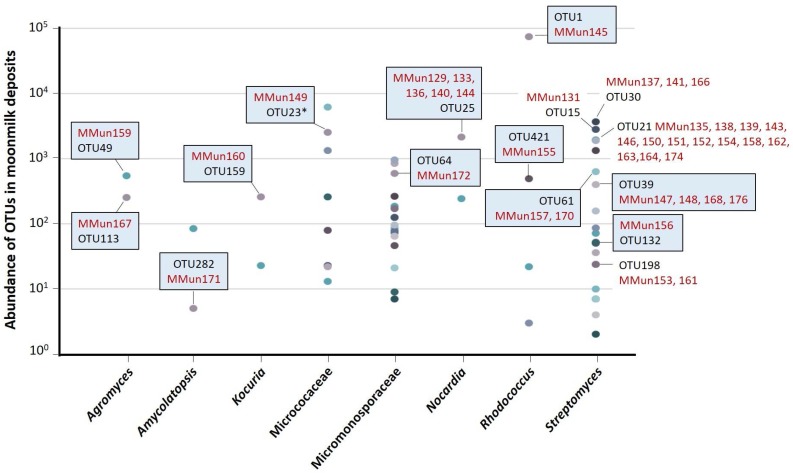
Membership of MMun strains isolated in this study to the actinobacterial operational taxonomic units (OTUs) identified by the high-throughput sequencing (HTS) metagenetic study. OTUs in blue boxes are those for which we isolated a first representative strain in this study. Note that for MMun149 and MMun172, the search of their associated OTU was performed at the family level (Micrococcaceae and Micromonosporaceae) and not at the genus level, as the HTS study [13] did not allow us to unambiguously assign the sequences of the 16S rRNA V6–V7 amplicons to the *Micrococcus* and *Micromonospora* genera. Symbol: *, due to the phylogenetic divergence of its V6–V7 region and based on the full sequence of its 16S rRNA gene (extracted from the genome sequence), MMun149 was manually affiliated to OTU23 even though its “closest” OTU in the tree was OTU141 (Figure S2).

An important question is “to which of the 243 actinobacterial OTUs (Table S1) identified by HTS [13] in the studied moonmilk deposits are the 40 new actinobacterial isolates associated”. In other words, did the isolation protocols used in this study enable the recovery of abundant and widespread strains or Actinobacteria with much more limited abundance and distribution in these speleothems (or both). For this purpose, we compared the 16S rRNA sequences (trimmed to the V6-V7 variable region) of the new isolates with the 16S rRNA amplicons (V6–V7 region) of the 243 actinobacterial OTUs obtained in our metagenetic study on DNA extracted from the same moonmilk deposits [13]. The phylogenetic tree positioning the 40 MMun strains (Table 1) with the 243 OTUs [13] is displayed in Supplementary Figures S1 and S2 and the results are summarized in Figure 5.

Figure 5 reveals that our study enabled the isolation of a strain (MMun145) associated with the most abundant OTU of the studied carbonate deposits—OTU1, which was assigned to the *Rhodococcus* genus and accounted for 16.45% (75,143 sequences) of the total number of 16S rRNA amplicon sequences obtained by our HTS analysis [13]. BLAST analysis of the full-length 16S rRNA sequence of MMun145 found *Rhodococcus* sp. strain MAK1 [30] as the closest hit, a strain isolated from diesel oil-contaminated soil and therefore possibly adapted to life in extreme environments. In another (and opposite) extreme case, MMun171 (*Amycolatopsis*) was associated with one of the less abundant OTUs—OTU282, which corresponded to only five 16S rRNA amplicon sequences identified (0.001%) in our metagenetic study [13]. This extremely rare Actinobacteria, only detected in collection point number 4 of the Grotte des Collemboles [13], has been able to grow in the ISP5 medium with separate autoclaving of the phosphate and agar components, as well as in media supplemented by iron and DFB (Table 1). 

The other isolated MMun strains belong to OTUs that stand between these two extreme ends, but generally, they belong to some of the most abundant OTUs of their respective genus/family (Figure 5). It must be noted that eight of the newly identified *Streptomyces* strains are associated with OTUs for which culturable representatives had not been isolated in our previous screening [12] (Figure 5). However, no culturable *Streptomyces* strains have been isolated from twelve of the 19 *Streptomyces* OTUs, which highlights how many moonmilk-dwelling species remain to be discovered. 

### 2.3. Evaluation of the Antibacterial Activity of the New Moonmilk Isolates

**Figure 6:**
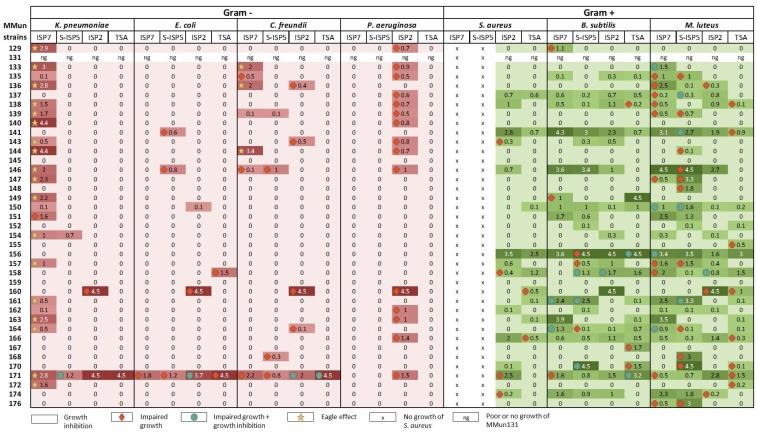
Heatmap illustrating antibacterial activities of the moonmilk actinobacteria isolated in this study. The size of the inhibition zone (in cm) is given for each strain.

**Figure 7:**
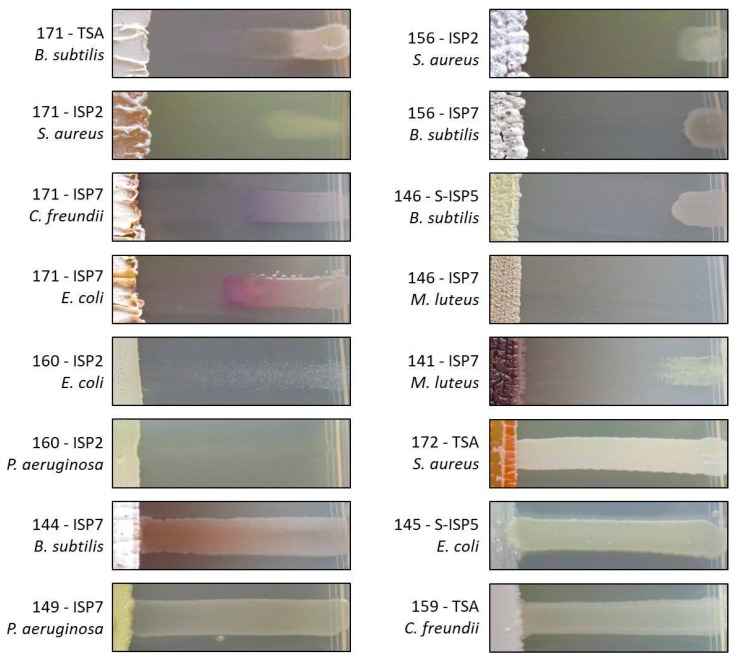
Selected representative examples of the cross-streak results. MMun strain numbers, media, and tested cultures are mentioned.

The potential to produce compounds with antimicrobial activity against Gram-positive and Gram-negative bacteria was evaluated for each strain via cross-streak assays (see the heatmap presented in Figure 6). Strains were streaked on ISP2, S-ISP5, ISP7, and TSA agar plates, and incubated for 14 days at 28 °C. Two types of antibacterial activities were recorded: (i) those that fully inhibited the growth (GI, growth inhibition) of the tested reference strains (see Figure 7 MMun156 against *B. subtilis* as a GI example) and (ii) those that allowed only partial growth (IG, impaired growth, see Figure 7 MMun160 against *E. coli* as an IG example). On several occasions, both GI and IG were observed (see Figure 7 MMun171 against *B. subtilis* as an example). 

**Figure 8:**
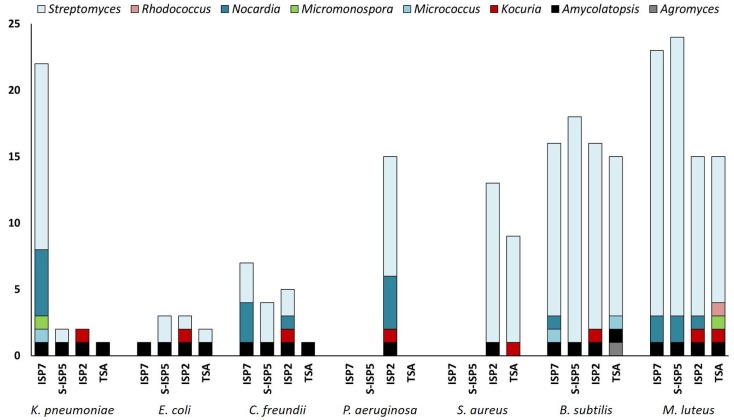
Number of strains with antibacterial activity, broken down by actinobacterial genus and test conditions.

As expected, much stronger overall inhibitory activities (GI and IG) were recorded against Gram-positive bacteria (~87% of the MMun strains) compared to those against Gram-negative bacteria (~59% of the MMun strains) (Figure 8). Two isolates were particularly active against the tested Gram-negative bacteria—MMun160 (*Kocuria*) and MMun171 (*Amycolatopsis*) (Figure 6 and Figure 7). However, these two strains exhibited very different activity patterns depending on the culture conditions: while MMun171 displayed strong inhibitory activity under all culture conditions, MM160 only secreted its antibiotic(s) when cultured in the ISP2 medium (Figure 6). 

Regarding the antibacterial activities against the Gram-positive strains, their growth was affected by more actinobacterial isolates and was overall more strongly inhibited (Figure 6 and Figure 7). Three moonmilk *Streptomyces* strains (MMun141, MMun146, and MMun156) were found to be particularly active, as they exhibited strong inhibitory activities against all tested Gram-positive species and under each culture condition tested (Figure 6 and examples shown in Figure 7). Interestingly, the same isolates showed virtually no inhibitory effect against Gram-negative bacteria. In addition, the moonmilk isolates most active against Gram-negative pathogens (MMun160 and MMun171) also displayed quite strong inhibitory profiles against Gram-positive strains.

In contrast, a few strains did not display any (or extremely weak) antibacterial activity, such as isolates MMun145 and MMun155 that belong to the *Rhodococcus* genus, the two *Agromyces* strains (MMun159 and MMun167), and the *Micromonospora* isolate MMun172 (Figure 6, Figure 7 and Figure 8). Similarly, the five *Nocardia* isolates did not secrete compounds with antibacterial activity under most conditions, except isolate MMun136, which displayed weak antibacterial activity against *M. luteus*. Therefore, while we managed to uncover conditions to isolate and grow these rare Actinobacteria, the conditions that enable production of their putative antibiotics remain undefined. 

**Figure 9:**
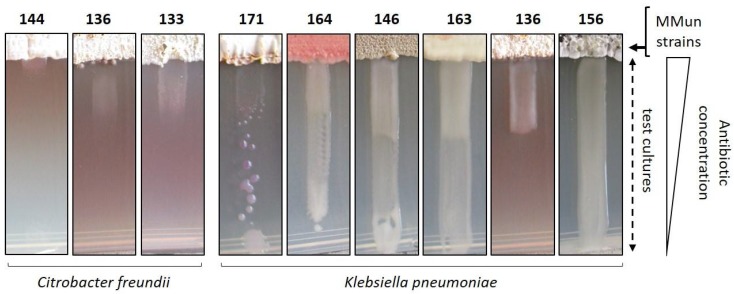
Illustration of the paradoxical zone phenomenon (or Eagle effect) observed with *Klebsiella pneumonia* and *Citrobacter freundii*. MMun156 was chosen as a control to show non-affected growth of *K. pneumoniae* on the ISP7 medium. All conditions where an Eagle effect was observed are marked with a “star” symbol in Figure 6.

Interestingly, unusual growth/susceptibility of *Klebsiella pneumonia* was frequently observed in the ISP7 medium, as reported in our first antibiotic activity screening assays [11]. Examples of different atypical growth responses are presented in Figure 9. In the depicted cases, we observed a non-linear response to the diffusion gradient of the secreted antibiotics, which has been described as the ‘Eagle effect’ (or paradoxical zone phenomenon) [31]. *K. pneumoniae* is indeed often able to grow near the actinobacterial strain, while its susceptibility to the secreted compounds is increased at a higher distance. Occasionally, the ‘Eagle effect’ response was also observed with *Citrobacter freundii*.

Finally, draft genome assemblies of 20 MMun isolates were mined for biosynthetic gene clusters (BGCs) in order to estimate their potential at producing natural compounds. More precisely, we examined the presence of BGCs containing polyketide synthases (PKSs, including PKS-I, PKS-II, and PKS-III) and NRPS domains, as described previously [12]. The evaluation of the genetic predisposition to produce natural compounds of isolates for which we obtained genome assemblies is summarized in Table 2. 

All investigated isolates encode in their genomes at least four and up to 34 NRPS domains. All strains (except MMun154 and MMun145) also possess multiple PKS-I (up to 21, MMun146) and PKS-II (up to 6, MMun131, 137, and 141) domains. PKS-IIIs were expectedly found less frequently (maximum 2), albeit 70% contained at least one BGC including a PKS-III domain. Interestingly, the highest number of BGCs was found in all isolated *Nocardia* strains, with MMun133 harboring the largest amount of biosynthetic cluster domains (45 in total). The fact that the strains that harbor the highest number of BGCs (Table 2) did not display any—or extremely weak or only Eagle-effect—antibacterial activity (Figure 6) suggests that if we indeed found conditions to isolate and grow them in synthetic media, we still have to discover the culture conditions that will trigger their secondary metabolism. 

## 3. Materials and Methods 

### 3.1. Preparation of Media and Moonmilk Suspension for Isolation of Rare Actinobacteria

Starch nitrate (SN) [32] and International Streptomyces Project (ISP) medium number 5 (ISP5) [33] were prepared in two different manners: either according to the standard protocols with all of the components autoclaved together (SN/ISP5) or with the agar and phosphate solution autoclaved separately and mixed before media pouring (S-SN/S-ISP5) in order to avoid hydrogen peroxide (H_2_O_2_) formation [23]. Media were supplemented with nalidixic acid (75 μg/mL) and nystatin (50 μg/mL) to suppress the growth of Gram-negative bacteria and fungi, respectively. Each medium was additionally supplemented with factors known or predicted to increase microbial growth, including: (i) 20 µM FeCl_3_; (ii) 1 µM of desferrioxamine B (Sigma-Aldrich, St. Louis, MO, USA); and (iii) 10 µl of 10 mg/mL bovine liver catalase (Sigma-Aldrich, St. Louis, MO, USA) (on the Whatman 3 MM paper disc (Sigma-Aldrich, St. Louis, MO, USA) in the center of the Petri dish) [23]. 

Isolation of Actinobacteria was performed by preparing a moonmilk suspension via the rehydration-centrifugation method according to Hop et al. (2011) [25]. Briefly, 0.45 g of freeze-dried moonmilk (0.15 g of each collection site—COL1, COL3, and COL4) was suspended in 50 mL of 0.01 M phosphate buffer (pH 7.0), vortexed for 5 min, and incubated for 90 min at 30 °C. Then, 8 mL of the upper part of the supernatant was transferred to a 50 mL conical centrifuge tube and centrifuged at room temperature for 10 min at 3000 rpm. After a 30-min incubation at 30 °C, 100 µL of serial dilutions (up to 10^−4^) were inoculated on the different solid media. All plates were incubated at 15 °C for about 2 months. The number of CFUs was evaluated after 16, 44, and 69 days of incubation. Among the 100 colonies selected from the culturable population, 42 isolates (named MMun) were successfully subcultured twice to obtain pure strains and stored both as 25% glycerol mycelium stock at −20 °C and in solid media at 4 °C. 

### 3.2. Isolation and Sequencing of Genomic DNA

Genomic DNA from MMun strains was extracted with the GenElute Bacterial Genomic DNA Kit (Sigma-Aldrich, St. Louis, MO, USA) according to the manufacturer’s instructions from the mycelium grown in liquid LB, ISP1, or ISP2 media at 28 °C. Genomic libraries of moonmilk isolates were constructed using the Nextera XT kit (Illumina, Inc., San Diego, CA, USA). Sequencing was carried out on the Illumina MiSeq platform with 2 × 300-bp read configuration. Complete genomes were assembled de novo from raw sequence data with SPAdes v3.6.2 [34] using the “careful” option, and the quality of the assemblies was subsequently assessed with QUAST v2.3 [35]. 

### 3.3. Phylogenetic Analyses of Actinobacterial Strains

The phylogenetic analysis of the moonmilk-derived strains shown in Figure 4 was based on either the full-length or the nearly full-length sequences of their 16S rRNA gene, which were either (i) recovered from the sequenced genomes (Table 2) or (ii) amplified using bacterial universal primers 8F and 1541R [36], as reported previously [27]. A multiple sequence alignment (1538 sites) was built with MUSCLE v3.8.31 [37] (default parameters) and filtered using the script ali2phylip.pl (from the Bio-MUST-Core software package; D. Baurain; https://metacpan.org/release/Bio-MUST-Core), so as to only keep sites with no missing character states. The filtered alignment (92 sequences × 1342 sites) was then submitted to phylogenetic inference using the rapid bootstrap analysis of RAxML v8.1.17 ([38]; 100 pseudoreplicates) under the model GTR + Γ_4_.

The V6–V7 regions of the 40 MMun strains were extracted from their 16S rRNA full-length sequences and combined to the corresponding regions of the 243 metagenetic OTUs to yield the tree shown in Supplementary Figure S1. Briefly, the final alignment was carefully crafted using a combination of automatic and manual approaches, including MAFFT v7.273 [39], Clustal Omega v1.1.0 [40], and the alignment editor (ed) of the MUST software package [41]. OTU403 was discarded because of its identity to OTU1, except for an aberrant five-terminal stretch. The optimized alignment (296 sites) was then filtered using ali2phylip.pl to remove sites present in <5% of the sequences, resulting in a final dataset of 282 sequences × 261 sites (0.12% of missing character states). Phylogenetic inference was carried out as for the full-length 16S rRNA genes above. The tree was first formatted in FigTree v1.4.21 (http://tree.bio.ed.ac.uk/software/figtree/) and then further arranged using Inkscape v0.91 (https://inkscape.org/). Patristic distances were then obtained using SeaView v4.5.4 [42] and the OTU closest to each MMun V6-V7 region was automatically extracted using a custom Perl script. Both the tree topology and the patristic distances were considered to affiliate MMun strains to metagenetic OTUs (Supplementary Figures S1 and S2).

The accession numbers of the 16S rRNA deposited in the GenBank database are listed in the Supplementary Table S2.

### 3.4. Antimicrobial Activities of Rare Moonmilk Actinobacterial Strains

Antibacterial activities of “MMun” strains of our collection were evaluated via cross-streak assays as described previously [11]. Each strain was inoculated from a mycelium stock as a single line in the center of the square plate and incubated for 14 days at 28 °C before cross-streaked with bacterial reference strains: *Escherichia coli* (ATCC 25922), *Pseudomonas aeruginosa* (ATCC 27853), *Citrobacter freundii* (ATCC 43864), *Klebsiella pneumoniae* (ATCC 13883), *Bacillus subtilis* (ATCC 19659), *Staphylococcus aureus* (ATCC 25923), and *Micrococcus luteus* (ATCC 9341).

### 3.5. Genome Mining for Gene Clusters Involved in Secondary Metabolite Production.

Genomes of 20 MMun strains were screened for NRPS and PKS genes, as described previously [11]. Only contigs ≥10 kb were considered and genes were identified as NRPS and PKS-I only when they displayed adenylation and acyltransferase domains, respectively.

## 4. Conclusions

Motivated by the results of our metagenetic study that had revealed a high diversity of the actinobacterial microbiome dwelling in the moonmilk deposits of the “Grotte des Collemboles” [13], we set up a series of protocols and culture conditions to isolate representatives of some of the most promising actinobacterial genera for the production of natural compounds. Remarkably, we isolated for the first time rare Actinobacteria belonging to the *Agromyces*, *Amycolatopsis*, *Kocuria*, *Micrococcus*, *Micromonospora*, *Nocardia,* and *Rhodococcus* genera, as well as additional new members of the *Streptomyces* genus. Remarkably, we succeeded to isolate for the first time from moonmilk deposits actinobacterial strains that belong to the *Micrococcus*, *Micromonospora*, and *Kocuria* genera. Another remarkable outcome of our research is the isolation of an *Amycolatopsis* strain that belongs to one of the less abundant OTUs identified in the studied moonmilk, accounting for less than 0.001% of the actinobacterial microbiome. This strain has also been revealed to display the highest propensity for producing antibacterial agents, both in terms of intensity and spectrum of activity.

Despite our success at isolating strains belonging to different actinobacterial genera, we however encountered a significant loss of the isolated colonies (58%) during our steps to obtain pure cultures. This most likely highlights the necessity of these strains to grow in the presence of specific growth factors of their environmental niche or an obligation to evolve in a mutualistic population with other moonmilk-dwelling bacteria. This is particularly problematic in bioprospecting, as the lost strains have probably been unable to grow as pure isolates because they are particularly (too) well-adapted to their unique natural habitat, and this adaptation might be a consequence of a unique specialized (secondary) metabolism. Therefore, we might have lost some of the most interesting strains in terms of potential producers of novel bioactive compounds.

In line with the conclusion mentioned above, though in this work we managed to isolate novel strains (and most likely novel species) thanks to our adapted protocols; the comparison of the 16S rRNA sequences of the new isolates with the 16S rRNA amplicons obtained in our metagenetic study [13] revealed the weak representativeness of our collection compared to Actinobacteria actually dwelling in the studied moonmilk deposits. Indeed, the identified 132 culturable strains are affiliated with 16 (6.5%) of the 243 actinobacterial OTUs identified from the DNA extracted from the moonmilk deposits. Considering that one single OTU can include lots of different species and strains (see OTU21 as example, Figure 4), this further emphasizes how much our collection is not exhaustively representative of the actinobacterial community inhabiting these carbonate deposits. 

Finally, finding novel species/strains is only the first step towards the discovery of novel natural compounds. The next step is to make them trigger their secondary metabolism, which is an equally challenging task. Indeed, even if some strains already display high antibacterial activity, many others remain metabolically silent under the tested culture conditions. This is the case for all *Nocardia* strains isolated in this study that were unable to display antibacterial activity but were revealed to possess the largest number of BGCs (up to 45). As most of our new moonmilk strains have been isolated using a series of “tips and tricks” to cultivate the unculturable, they are unlikely to grow in many different media, which limits the culture conditions to be tested to awaken their secondary metabolism. Mining their genomes to unveil the triggers and cues of their secondary metabolism is a priority of our ongoing research [43]. 

## Figures and Tables

**Table 1:** Moonmilk strains isolated using the RC method and according to selective media.

MMun Strains	Family	Closest Match (BLAST)	16S rRNA Ident. %; gaps	Isolation Medium	Phylotype	OTU
129 ♣	Nocardiaceae	*Nocardia* sp. strain 2M-SSA4	99.40; 1	S-SN + Fe + DFB	-	25
130 ♣	Paenibacillaceae	*Paenibacillus* sp. DSL09-3	98.96; 1	S-SN + Fe + DFB	-	na
131 ♣	Streptomycetaceae	*Streptomyces* sp. 2323.1	99.61; 0	S-SN + Fe + DFB	XXXII	15
133 ♣	Nocardiaceae	*Nocardia* sp. strain 2M-SSA4	99.14; 3	S-SN + Fe + DFB	-	25
135 ♣	Streptomycetaceae	*Streptomyces* sp. MM63	100; 0	SN + Fe	IV *	21
136 ♣	Nocardiaceae	*Nocardia soli* strain DSM 44488	100; 0	SN + Fe	-	25
137 ♣	Streptomycetaceae	*Streptomyces* sp. MM82	99.08; 2	SN + DFB	XXXIII	30
138 ♣	Streptomycetaceae	*Streptomyces* sp. MM63	100; 0	SN	IV *	21
139 ♣	Streptomycetaceae	*Streptomyces* sp. MM63	100; 0	SN	IV *	21
140 ♣	Nocardiaceae	*Nocardia* sp. strain 2M-SSA4	99.54; 1	SN	-	25
141 ♣	Streptomycetaceae	*Streptomyces* sp. MM82	99.15; 2	SN	XXXIV	30
142 ♣	Rhodobacteraceae	Uncult. *Paracoccus* sp. clone 54	99.93; 0	SN + catalase	-	na
143 ♣	Streptomycetaceae	*Streptomyces lunaelactis* strain MM126	100; 0	SN + catalase	X *	21
144 ♣	Nocardiaceae	*Nocardia* soli strain Y48	99.34; 0	SN + catalase	-	25
145 ♣	Nocardiaceae	*Rhodococcus* sp. MTM3W5.2	99.80; 1	SN + catalase	-	1
146 ♣	Streptomycetaceae	*Streptomyces* sp. MM44	100; 0	S-SN + DFB	XXIX *	21
147 ♣	Streptomycetaceae	*Streptomyces xiamenensis* strain 318	98.69; 8	S-SN + DFB	XXXV	39
148 ♣	Streptomycetaceae	*Streptomyces xiamenensis* strain 318	98.69; 8	S-SN + DFB	XXXV	39
149	Micrococcaceae	*Micrococcus* sp. 3455	100; 0	SN	-	23
150 ♣	Streptomycetaceae	*Streptomyces* sp. MM63	100; 0	SN	IV *	21
151 ♣	Streptomycetaceae	*Streptomyces* sp. MM110	100; 0	SN	XXI *	21
152	Streptomycetaceae	*Streptomyces lunaelactis* strain MM126	100; 0	SN + Fe	X *	21
153	Streptomycetaceae	*Streptomyces* sp. MM18	99.93; 1	SN + DFB	I * (lost)	198
154 ♣	Streptomycetaceae	*Streptomyces* sp. ND04-1H	99.93; 0	SN	XXXVI	21
155	Nocardiaceae	*Rhodococcus* sp. strain MAK1	99.86; 2	SN	-	421
156	Streptomycetaceae	*Streptomyces* sp. 1C-HV8	99.86; 2	S-SN + Fe + DFB	XXXVII	132
157 ♣	Streptomycetaceae	*Streptomyces* sp. 2M-TWYE1	99.47; 0	SN + catalase	XXXVIII	61
158	Streptomycetaceae	*Streptomyces* sp. SpC090624KE_06	99.51; 0	S-SN + Fe + DFB	XXXIX	21
159	Microbacteriaceae	*Agromyces* sp. strain 4K403B	97.88; 14	S-SN + DFB	-	49
160	Micrococcaceae	*Kocuria rhizophila* strain R-42745	99.93; 0	S-SN	-	159
161	Streptomycetaceae	*Streptomyces* sp. MM18	100; 0	S-SN + Fe + DFB	I *	198
162	Streptomycetaceae	*Streptomyces* sp. MM63	99.93; 1	S-SN + Fe + DFB	IV *	21
163	Streptomycetaceae	*Streptomyces* sp. MM63	100; 0	S-SN	IV *	21
164	Streptomycetaceae	*Streptomyces* sp. MM63	100; 0	S-SN	IV *	21
166	Streptomycetaceae	*Streptomyces* sp. strain A301	99.23; 3	S-ISP5 + Fe + DFB	XXXIV	30
167	Microbacteriaceae	*Agromyces cerinus* strain DSM 8595	99.58; 0	ISP5 + catalase	-	113
168	Streptomycetaceae	*Streptomyces* sp. 28a-5-1	98.60; 8	ISP5 + catalase	XXXV	39
170	Streptomycetaceae	*Streptomyces* sp. 2C-SSA16-1	99.64; 0	S-ISP5 + Fe + DFB	XL	61
171	Pseudonocardiaceae	*Amycolatopsis* sp. CA11	98.25; 16	S-ISP5 + Fe + DFB	-	282
172	Micromonosporaceae	*Micromonospora maoerensis* strain NEAU-MES19	99.93; 0	S-ISP5 + Fe + DFB	-	64
174	Streptomycetaceae	*Streptomyces* sp. MM110	100; 0	ISP5 + catalase	XXI *	21
176	Streptomycetaceae	*Streptomyces* sp. 28a-5-1	98.67; 7	S-ISP5	XXXV	39

Numbers in the phylotype column have been deduced from the phylogenetic tree presented in Figure 4 and based on our previous screening [11]. The last column indicates to which OTU identified in our metagenetic study [13] the MMun strains are affiliated (according to the topology and patristic distances of the tree in Supplementary Figures S1 and S2). Abbreviations: S- before SN and ISP5, indicates that media were prepared by autoclaving of agar and phosphate separate from other media components; DFB, desferrioxamine B; na, not Actinobacteria; *, phylotypes described in our previous study [12]; lost, culture from mycelium stocks of MMun153 are no longer possible, and this strain is now considered as lost; and ♣, MMun isolates for which the genome has been sequenced.

**Table 2:** Number of biosynthetic gene clusters (BGCs) based on genome mining of 22 MMun strains.

MMun	PKS-I	PKS-II	PKS-III	NRPS	Total	Genus
129	2	2	1	34	39	*Nocardia*
131	2	6	1	27	36	*Streptomyces*
133	7	4	1	33	45	*Nocardia*
135	10	4	1	16	31	*Streptomyces*
136	6	2	1	31	40	*Nocardia*
137	9	6	0	6	21	*Streptomyces*
138	11	4	1	16	32	*Streptomyces*
139	4	4	0	13	21	*Streptomyces*
140	2	2	1	32	37	*Nocardia*
141	9	6	0	6	21	*Streptomyces*
143	6	4	1	9	20	*Streptomyces*
144	4	2	2	31	39	*Nocardia*
145	5	0	0	12	17	*Rhodococcus*
146	21	4	0	9	34	*Streptomyces*
147	10	2	1	4	17	*Streptomyces*
148	10	2	1	4	17	*Streptomyces*
150	12	4	1	14	31	*Streptomyces*
151	4	4	0	22	30	*Streptomyces*
154	0	4	1	10	15	*Streptomyces*
157	12	4	0	9	25	*Streptomyces*

## Supplementary Materials

The following are available online at http://www.mdpi.com/2079-6382/7/2/28/s1: Figure S1: Phylogenetic tree of newly isolated moonmilk-dwelling Actinobacteria strains based on the V6–V7 region of the 16S (SSU) rRNA gene; Figure S2: Closest OTUs (V6–V7 regions) for the 40 MMun strains; Table S1: OTUs and their abundance in moonmilk deposits of the “Grotte des Collemboles”; Table S2: Accession number of 16S rRNA sequences of MMun strains. Please provide the Table S2 that can be edited and put Figure S1 and Tables S1 and S2 into Word file.

## References

[1] Hill P., Heberlig G.W., Boddy C.N. (2017). Sampling Terrestrial Environments for Bacterial Polyketides. Molecules.

[2] Goodfellow M., Fiedler H.-P. (2010). A guide to successful bioprospecting: Informed by actinobacterial systematics. Antonie Leeuwenhoek.

[3] World Health Organization (WHO) (2014). Antimicrobial Resistance: Global Report on Surveillance 2014.

[4] Hutchinson G.E. (1961). The Paradox of the Plankton. Am. Nat..

[5] Bhullar K., Waglechner N., Pawlowski A., Koteva K., Banks E.D., Johnston M.D., Barton H.A., Wright G.D. (2012). Antibiotic resistance is prevalent in an isolated cave microbiome. PLoS ONE.

[6] Lavoie K., Ruhumbika T., Bawa A., Whitney A., de Ondarza J. (2017). High levels of antibiotic resistance but no antibiotic production detected along a gypsum gradient in great Onyx Cave, KY, USA. Diversity.

[7] Hopwood D.A. (2007). Streptomyces in Nature and Medicine: The Antibiotic Makers.

[8] Dhami N.K., Mukherjee A., Watkin E.L.J. (2018). Microbial Diversity and Mineralogical-Mechanical Properties of Calcitic Cave Speleothems in Natural and in Vitro Biomineralization Conditions. Front. Microbiol..

[9] Portillo M.C., Gonzalez J.M. (2011). Moonmilk Deposits Originate from Specific Bacterial Communities in Altamira Cave (Spain). Microb. Ecol..

[10] Rooney D.C., Hutchens E., Clipson N., Baldini J., McDermott F. (2010). Microbial Community Diversity of Moonmilk Deposits at Ballynamintra Cave, Co. Waterford, Ireland. Microb. Ecol..

[11] Maciejewska M., Adam D., Naômé A., Martinet L., Tenconi E., Całusińska M., Delfosse P., Hanikenne M., Baurain D., Compère P. (2017). Assessment of the Potential Role of Streptomyces in Cave Moonmilk Formation. Front. Microbiol..

[12] Maciejewska M., Adam D., Martinet L., Naômé A., Całusińska M., Delfosse P., Carnol M., Barton H.A., Hayette M.-P., Smargiasso N. (2016). A Phenotypic and Genotypic Analysis of the Antimicrobial Potential of Cultivable Streptomyces Isolated from Cave Moonmilk Deposits. Front. Microbiol..

[13] Maciejewska M., Calusinska M., Cornet L., Adam D., Pessi I.S., Malchair S., Delfosse P., Baurain D., Barton H.A., Carnol M. (2018). High-throughput sequencing analysis of the actinobacterial spatial diversity in moonmilk deposits. Antibiotics.

[14] Oliver J.D. (2005). The viable but nonculturable state in bacteria. J. Microbiol..

[15] Staley J.T., Konopka A. (1985). Measurement of in situ activities of nonphotosynthetic microorganisms in aquatic and terrestrial habitats. Annu. Rev. Microbiol..

[16] Hug L., Baker B., Anantharaman K., Brown C., Probst A., Castelle C., Butterfield C., Hernsdorf A., Amano Y., Ise K. (2016). A new view of the tree of life. Nat. Microbiol..

[17] Button D.K., Schut F., Quang P., Martin R., Robertson B.R. (1993). Viability and isolation of marine bacteria by dilution culture: Theory, procedures, and initial results. Appl. Environ. Microbiol..

[18] D’Onofrio A., Crawford J.M., Stewart E.J., Witt K., Gavrish E., Epstein S., Clardy J., Lewis K. (2010). Siderophores from Neighboring Organisms Promote the Growth of Uncultured Bacteria. Chem. Biol..

[19] Bruns A., Cypionka H., Overmann J. (2002). Cyclic AMP and acyl homoserine lactones increase the cultivation efficiency of heterotrophic bacteria from the central Baltic Sea. Appl. Environ. Microbiol..

[20] Sait M., Hugenholtz P., Janssen P.H. (2002). Cultivation of globally distributed soil bacteria from phylogenetic lineages previously only detected in cultivation-independent surveys. Environ. Microbiol..

[21] Nichols D., Cahoon N., Trakhtenberg E.M., Pham L., Mehta A., Belanger A., Kanigan T., Lewis K., Epstein S.S. (2010). Use of Ichip for High-Throughput In Situ Cultivation of “Uncultivable” Microbial Species. Appl. Environ. Microbiol..

[22] Tanaka T., Kawasaki K., Daimon S., Kitagawa W., Yamamoto K., Tamaki H., Tanaka M., Nakatsu C.H., Kamagata Y. (2014). A hidden pitfall in the preparation of agar media undermines microorganism cultivability. Appl. Environ. Microbiol..

[23] Kawasaki K., Kamagata Y. (2017). Phosphate-Catalyzed Hydrogen Peroxide Formation from Agar, Gellan, and κ-Carrageenan and Recovery of Microbial Cultivability via Catalase and Pyruvate. Appl. Environ. Microbiol..

[24] Jiang C.-Y., Dong L., Zhao J.-K., Hu X., Shen C., Qiao Y., Zhang X., Wang Y., Ismagilov R.F., Liu S.-J. (2016). High-Throughput Single-Cell Cultivation on Microfluidic Streak Plates. Appl. Environ. Microbiol..

[25] Hop D.V., Sakiyama Y., Binh C.T.T., Otoguro M., Hang D.T., Miyadoh S., Luong D.T., Ando K. (2011). Taxonomic and ecological studies of actinomycetes from Vietnam: Isolation and genus-level diversity. J. Antibiot..

[26] Otoguro M., Hayakawa M., Yamazaki T., Iimura Y. (2001). An integrated method for the enrichment and selective isolation of Actinokineospora spp. in soil and plant litter. J. Appl. Microbiol..

[27] Hayakawa M., Otoguro M., Takeuchi T., Yamazaki T., Iimura Y. (2000). Application of a method incorporating differential centrifugation for selective isolation of motile actinomycetes in soil and plant litter. Antonie Leeuwenhoek.

[28] Arias A.A., Lambert S., Martinet L., Adam D., Tenconi E., Hayette M.-P., Ongena M., Rigali S. (2015). Growth of desferrioxamine-deficient Streptomyces mutants through xenosiderophore piracy of airborne fungal contaminations. FEMS Microbiol. Ecol..

[29] Maciejewska M., Pessi I.S., Arguelles-Arias A., Noirfalise P., Luis G., Ongena M., Barton H., Carnol M., Rigali S. (2015). Streptomyces lunaelactis sp. nov., a novel ferroverdin A-producing Streptomyces species isolated from a moonmilk speleothem. Antonie Leeuwenhoek.

[30] Kis Á.E., Laczi K., Zsíros S., Kós P., Tengölics R., Bounedjoum N., Kovács T., Rákhely G., Perei K. (2017). Characterization of the Rhodococcus sp. MK1 strain and its pilot application for bioremediation of diesel oil-contaminated soil. Acta Microbiol. Immunol. Hung..

[31] Eagle H., Musselman A.D. (1948). The rate of bactericidal action of penicillin in vitro as a function of its concentrations, and its paradoxically reduced activity at high concentrations against certain organisms. J. Exp. Med..

[32] Waksman S., Lechevalier H. (1961). The Actinomycetales, Classification, Identification and Description of Genera and Species.

[33] Shirling E.B., Gottlieb D. (1966). Methods for characterization of Streptomyces species. Int. J. Syst. Bacteriol..

[34] Bankevich A., Nurk S., Antipov D., Gurevich A.A., Dvorkin M., Kulikov A.S., Lesin V.M., Nikolenko S.I., Pham S., Prjibelski A.D. (2012). SPAdes: A new genome assembly algorithm and its applications to single-cell sequencing. J. Comput. Biol..

[35] Gurevich A., Saveliev V., Vyahhi N., Tesler G. (2013). QUAST: Quality assessment tool for genome assemblies. Bioinformatics.

[36] Onstott T.C., McGown D.J., Bakermans C., Ruskeeniemi T., Ahonen L., Telling J., Soffientino B., Pfiffner S.M., Sherwood-Lollar B., Frape S. (2009). Microbial communities in subpermafrost saline fracture water at the Lupin Au mine, Nunavut, Canada. Microb. Ecol..

[37] Edgar R.C. (2004). MUSCLE: Multiple sequence alignment with high accuracy and high throughput. Nucleic Acids Res..

[38] Stamatakis A. (2014). RAxML version 8: A tool for phylogenetic analysis and post-analysis of large phylogenies. Bioinformatics.

[39] Katoh K., Standley D.M. (2013). MAFFT multiple sequence alignment software version 7: Improvements in performance and usability. Mol. Biol. Evol..

[40] Sievers F., Wilm A., Dineen D., Gibson T.J., Karplus K., Li W., Lopez R., McWilliam H., Remmert M., Söding J. (2011). Fast, scalable generation of high-quality protein multiple sequence alignments using Clustal Omega. Mol. Syst. Biol..

[41] Philippe H. (1993). MUST, a computer package of Management Utilities for Sequences and Trees. Nucleic Acids Res..

[42] Gouy M., Guindon S., Gascuel O. (2010). SeaView version 4: A multiplatform graphical user interface for sequence alignment and phylogenetic tree building. Mol. Biol. Evol..

[43] Rigali S., Anderssen S., Naômé A., van Wezel G.P. (2018). Cracking the Regulatory Code of Biosynthetic Gene Clusters as a Strategy for Natural Product Discovery. Biochem. Pharmacol..

