# Supplementary material for: Isolation, Characterization, and Antibacterial Activity of Hard-to-Culture Actinobacteria from Cave Moonmilk Deposits
Source: Antibiotics (Basel). 2018 Mar 22;7(2):28. doi: 10.3390/antibiotics7020028 (PMC6023089; doi:10.3390/antibiotics7020028)
Supplement: Supplementary file 1 [file antibiotics-07-00028-s001.zip › Supplementary Table S1.docx]

**Table S1. OTUs and their abundance in moonmilk deposits of the “Grotte des Collemboles”**

| **OTU** | **COL1** |  | **COL3** |  | **COL4** |  | **Sum of seq** | **%** | **Class** | **Order** | **Family** | **Genus** |
| --- | --- | --- | --- | --- | --- | --- | --- | --- | --- | --- | --- | --- |
| **OTU1** | 54789 | 38.28 | 16390 | 9.68 | 3964 | 2.74 | 75143 | 16.45 | Actinobacteria | Corynebacteriales | Nocardiaceae | Rhodococcus |
| **OTU10** | 1154 | 0.81 | 484 | 0.29 | 2433 | 1.68 | 4071 | 0.89 | Acidimicrobiia | Acidimicrobiales | Acidimicrobiaceae | uncultured |
| **OTU101** | 104 | 0.07 | 46 | 0.03 | 13 | 0.01 | 163 | 0.04 | Actinobacteria | Micrococcales | Microbacteriaceae | unclassified |
| **OTU102** | 0 | 0.00 | 98 | 0.06 | 0 | 0.00 | 98 | 0.02 | Actinobacteria | Streptosporangiales | Streptosporangiaceae | unclassified |
| **OTU103** | 87 | 0.06 | 0 | 0.00 | 0 | 0.00 | 87 | 0.02 | Actinobacteria | Corynebacteriales | Nocardiaceae | unclassified |
| **OTU104** | 86 | 0.06 | 0 | 0.00 | 0 | 0.00 | 86 | 0.02 | Actinobacteria | Pseudonocardiales | Pseudonocardiaceae | Amycolatopsis |
| **OTU105** | 125 | 0.09 | 14 | 0.01 | 88 | 0.06 | 227 | 0.05 | Acidimicrobiia | Acidimicrobiales | uncultured | uncultured |
| **OTU108** | 57 | 0.04 | 39 | 0.02 | 53 | 0.04 | 149 | 0.03 | Actinobacteria | Propionibacteriales | Nocardioidaceae | Nocardioides |
| **OTU109** | 0 | 0.00 | 0 | 0.00 | 21 | 0.01 | 21 | 0.00 | Actinobacteria | Micrococcales | Promicromonosporaceae | Promicromonospora |
| **OTU11** | 538 | 0.38 | 58 | 0.03 | 2490 | 1.72 | 3086 | 0.68 | Acidimicrobiia | Acidimicrobiales | uncultured | uncultured |
| **OTU110** | 119 | 0.08 | 38 | 0.02 | 8 | 0.01 | 165 | 0.04 | unclasiffied | unclassified | unclassified | uncultured |
| **OTU111** | 21 | 0.01 | 28 | 0.02 | 77 | 0.05 | 126 | 0.03 | Actinobacteria | Micromonosporales | Micromonosporaceae | Catellatospora |
| **OTU112** | 69 | 0.05 | 0 | 0.00 | 6 | 0.00 | 75 | 0.02 | Actinobacteria | Corynebacteriales | Mycobacteriaceae | unclassified |
| **OTU113** | 142 | 0.10 | 27 | 0.02 | 86 | 0.06 | 255 | 0.06 | Actinobacteria | Micrococcales | Microbacteriaceae | Agromyces |
| **OTU114** | 70 | 0.05 | 0 | 0.00 | 3 | 0.00 | 73 | 0.02 | Actinobacteria | Micromonosporales | Micromonosporaceae | unclassified |
| **OTU116** | 101 | 0.07 | 15 | 0.01 | 0 | 0.00 | 116 | 0.03 | Actinobacteria | unclasiffied | uncultured | uncultured |
| **OTU118** | 61 | 0.04 | 0 | 0.00 | 19 | 0.01 | 80 | 0.02 | Actinobacteria | Streptosporangiales | Streptosporangiaceae | Streptosporangium |
| **OTU119** | 0 | 0.00 | 0 | 0.00 | 21 | 0.01 | 21 | 0.00 | unclasiffied | unclassified | unclassified | uncultured |
| **OTU12** | 4252 | 2.97 | 11180 | 6.61 | 2623 | 1.82 | 18055 | 3.95 | Actinobacteria | Pseudonocardiales | Pseudonocardiaceae | uncultured |
| **OTU121** | 78 | 0.05 | 0 | 0.00 | 0 | 0.00 | 78 | 0.02 | Actinobacteria | Corynebacteriales | Corynebacteriaceae | uncultured |
| **OTU122** | 0 | 0.00 | 21 | 0.01 | 24 | 0.02 | 45 | 0.01 | Actinobacteria | Frankiales | Frankiaceae | Jatrophihabitans |
| **OTU123** | 27 | 0.02 | 0 | 0.00 | 24 | 0.02 | 51 | 0.01 | Actinobacteria | Propionibacteriales | Nocardioidaceae | Nocardioides |
| **OTU125** | 107 | 0.07 | 0 | 0.00 | 31 | 0.02 | 138 | 0.03 | Acidimicrobiia | Acidimicrobiales | Iamiaceae | Iamia |
| **OTU126** | 70 | 0.05 | 0 | 0.00 | 16 | 0.01 | 86 | 0.02 | Actinobacteria | Streptomycetales | Streptomycetaceae | Streptomyces |
| **OTU129** | 66 | 0.05 | 0 | 0.00 | 12 | 0.01 | 78 | 0.02 | Actinobacteria | Micromonosporales | Micromonosporaceae | unclassified |
| **OTU13** | 658 | 0.46 | 8306 | 4.91 | 5281 | 3.65 | 14245 | 3.12 | Actinobacteria | Pseudonocardiales | Pseudonocardiaceae | Pseudonocardia |
| **OTU130** | 0 | 0.00 | 0 | 0.00 | 23 | 0.02 | 23 | 0.01 | Acidimicrobiia | Acidimicrobiales | uncultured | uncultured |
| **OTU131** | 73 | 0.05 | 0 | 0.00 | 25 | 0.02 | 98 | 0.02 | Actinobacteria | Propionibacteriales | Nocardioidaceae | Aeromicrobium |
| **OTU132** | 46 | 0.03 | 0 | 0.00 | 4 | 0.00 | 50 | 0.01 | Actinobacteria | Streptomycetales | Streptomycetaceae | Streptomyces |
| **OTU133** | 515 | 0.36 | 55 | 0.03 | 92 | 0.06 | 662 | 0.14 | Acidimicrobiia | Acidimicrobiales | uncultured | uncultured |
| **OTU134** | 0 | 0.00 | 0 | 0.00 | 30 | 0.02 | 30 | 0.01 | unclasiffied | unclassified | unclassified | uncultured |
| **OTU136** | 58 | 0.04 | 0 | 0.00 | 0 | 0.00 | 58 | 0.01 | Actinobacteria | Actinomycetales | Actinomycetaceae | Actinomyces |
| **OTU137** | 56 | 0.04 | 0 | 0.00 | 1 | 0.00 | 57 | 0.01 | Actinobacteria | unclasiffied | uncultured | uncultured |
| **OTU138** | 226 | 0.16 | 16 | 0.01 | 53 | 0.04 | 295 | 0.06 | Actinobacteria | Corynebacteriales | Mycobacteriaceae | unclassified |
| **OTU139** | 59 | 0.04 | 0 | 0.00 | 3 | 0.00 | 62 | 0.01 | Actinobacteria | Streptomycetales | Streptomycetaceae | unclassified |
| **OTU14** | 771 | 0.54 | 522 | 0.31 | 2497 | 1.73 | 3790 | 0.83 | Actinobacteria | Frankiales | Sporichthyaceae | Sporichthya |
| **OTU140** | 60 | 0.04 | 0 | 0.00 | 4 | 0.00 | 64 | 0.01 | Acidimicrobiia | Acidimicrobiales | uncultured | uncultured |
| **OTU141** | 78 | 0.05 | 1 | 0.00 | 6 | 0.00 | 85 | 0.02 | Actinobacteria | Actinomycetales | Actinomycetaceae | Actinomyces |
| **OTU142** | 1027 | 0.72 | 3239 | 1.91 | 3396 | 2.35 | 7662 | 1.68 | Actinobacteria | Pseudonocardiales | Pseudonocardiaceae | unclassified |
| **OTU143** | 57 | 0.04 | 0 | 0.00 | 0 | 0.00 | 57 | 0.01 | Actinobacteria | Propionibacteriales | Propionibacteriaceae | Propionibacterium |
| **OTU144** | 0 | 0.00 | 0 | 0.00 | 24 | 0.02 | 24 | 0.01 | Acidimicrobiia | Acidimicrobiales | Acidimicrobiaceae | uncultured |
| **OTU145** | 180 | 0.13 | 35 | 0.02 | 4 | 0.00 | 219 | 0.05 | Actinobacteria | Corynebacteriales | Corynebacteriaceae | unclassified |
| **OTU147** | 52 | 0.04 | 0 | 0.00 | 7 | 0.00 | 59 | 0.01 | Acidimicrobiia | Acidimicrobiales | uncultured | uncultured |
| **OTU148** | 76 | 0.05 | 0 | 0.00 | 0 | 0.00 | 76 | 0.02 | Actinobacteria | Propionibacteriales | Nocardioidaceae | Nocardioides |
| **OTU149** | 0 | 0.00 | 51 | 0.03 | 0 | 0.00 | 51 | 0.01 | Actinobacteria | Streptomycetales | Streptomycetaceae | Streptomyces |
| **OTU15** | 431 | 0.30 | 292 | 0.17 | 2132 | 1.48 | 2855 | 0.62 | Actinobacteria | Streptomycetales | Streptomycetaceae | Streptomyces |
| **OTU150** | 0 | 0.00 | 0 | 0.00 | 17 | 0.01 | 17 | 0.00 | Actinobacteria | Corynebacteriales | unclassified | unclassified |
| **OTU151** | 54 | 0.04 | 0 | 0.00 | 0 | 0.00 | 54 | 0.01 | Actinobacteria | Actinomycetales | Actinomycetaceae | Actinomyces |
| **OTU152** | 0 | 0.00 | 9 | 0.01 | 14 | 0.01 | 23 | 0.01 | Actinobacteria | Propionibacteriales | Nocardioidaceae | Nocardioides |
| **OTU155** | 0 | 0.00 | 42 | 0.02 | 4 | 0.00 | 46 | 0.01 | Actinobacteria | Micromonosporales | Micromonosporaceae | Catellatospora |
| **OTU156** | 0 | 0.00 | 7 | 0.00 | 19 | 0.01 | 26 | 0.01 | Acidimicrobiia | Acidimicrobiales | uncultured | uncultured |
| **OTU157** | 177 | 0.12 | 388 | 0.23 | 477 | 0.33 | 1042 | 0.23 | Acidimicrobiia | Acidimicrobiales | uncultured | uncultured |
| **OTU158** | 0 | 0.00 | 22 | 0.01 | 6 | 0.00 | 28 | 0.01 | Actinobacteria | Corynebacteriales | Gordoniaceae | Millisia |
| **OTU159** | 261 | 0.18 | 0 | 0.00 | 0 | 0.00 | 261 | 0.06 | Actinobacteria | Micrococcales | Micrococcaceae | Kocuria |
| **OTU16** | 348 | 0.24 | 869 | 0.51 | 1600 | 1.11 | 2817 | 0.62 | Acidimicrobiia | Acidimicrobiales | uncultured | uncultured |
| **OTU161** | 0 | 0.00 | 0 | 0.00 | 10 | 0.01 | 10 | 0.00 | Actinobacteria | unclasiffied | unclassified | unclassified |
| **OTU162** | 0 | 0.00 | 0 | 0.00 | 17 | 0.01 | 17 | 0.00 | unclasiffied | unclassified | unclassified | unclassified |
| **OTU163** | 0 | 0.00 | 0 | 0.00 | 16 | 0.01 | 16 | 0.00 | Actinobacteria | Acidothermales | Acidothermaceae | Acidothermus |
| **OTU166** | 0 | 0.00 | 0 | 0.00 | 12 | 0.01 | 12 | 0.00 | Actinobacteria | Corynebacteriales | unclassified | unclassified |
| **OTU167** | 45 | 0.03 | 0 | 0.00 | 0 | 0.00 | 45 | 0.01 | unclasiffied | unclassified | unclassified | uncultured |
| **OTU168** | 44 | 0.03 | 0 | 0.00 | 8 | 0.01 | 52 | 0.01 | Actinobacteria | Kineosporiales | Kineosporiaceae | Kineosporia |
| **OTU169** | 51 | 0.04 | 28 | 0.02 | 17 | 0.01 | 96 | 0.02 | Actinobacteria | Micromonosporales | Micromonosporaceae | uncultured |
| **OTU17** | 63 | 0.04 | 13 | 0.01 | 1360 | 0.94 | 1436 | 0.31 | Acidimicrobiia | Acidimicrobiales | uncultured | uncultured |
| **OTU170** | 0 | 0.00 | 0 | 0.00 | 36 | 0.02 | 36 | 0.01 | Actinobacteria | Streptomycetales | Streptomycetaceae | Streptomyces |
| **OTU171** | 0 | 0.00 | 0 | 0.00 | 18 | 0.01 | 18 | 0.00 | Acidimicrobiia | Acidimicrobiales | uncultured | uncultured |
| **OTU172** | 0 | 0.00 | 0 | 0.00 | 10 | 0.01 | 10 | 0.00 | Actinobacteria | Streptomycetales | Streptomycetaceae | Streptomyces |
| **OTU173** | 0 | 0.00 | 7 | 0.00 | 6 | 0.00 | 13 | 0.00 | Actinobacteria | Streptosporangiales | Streptosporangiaceae | unclassified |
| **OTU176** | 87 | 0.06 | 0 | 0.00 | 1577 | 1.09 | 1664 | 0.36 | unclasiffied | unclassified | unclassified | uncultured |
| **OTU177** | 28 | 0.02 | 0 | 0.00 | 0 | 0.00 | 28 | 0.01 | Actinobacteria | Actinomycetales | Actinomycetaceae | Actinomyces |
| **OTU179** | 0 | 0.00 | 0 | 0.00 | 11 | 0.01 | 11 | 0.00 | Actinobacteria | Micrococcales | Dermacoccaceae | Dermacoccus |
| **OTU18** | 688 | 0.48 | 198 | 0.12 | 1498 | 1.04 | 2384 | 0.52 | Acidimicrobiia | Acidimicrobiales | uncultured | uncultured |
| **OTU180** | 0 | 0.00 | 0 | 0.00 | 11 | 0.01 | 11 | 0.00 | Actinobacteria | Acidothermales | Acidothermaceae | uncultured |
| **OTU182** | 29 | 0.02 | 0 | 0.00 | 0 | 0.00 | 29 | 0.01 | Actinobacteria | Actinomycetales | Actinomycetaceae | Actinomyces |
| **OTU183** | 0 | 0.00 | 0 | 0.00 | 8 | 0.01 | 8 | 0.00 | Actinobacteria | Glycomycetales | Glycomycetaceae | Stackebrandtia |
| **OTU184** | 0 | 0.00 | 24 | 0.01 | 34 | 0.02 | 58 | 0.01 | Actinobacteria | Geodermatophilales | Geodermatophilaceae | uncultured |
| **OTU185** | 0 | 0.00 | 14 | 0.01 | 10 | 0.01 | 24 | 0.01 | Actinobacteria | Streptomycetales | Streptomycetaceae | Streptomyces |
| **OTU188** | 0 | 0.00 | 0 | 0.00 | 8 | 0.01 | 8 | 0.00 | Acidimicrobiia | Acidimicrobiales | uncultured | uncultured |
| **OTU19** | 2952 | 2.06 | 874 | 0.52 | 2388 | 1.65 | 6214 | 1.36 | Actinobacteria | Micrococcales | Micrococcaceae | Arthrobacter |
| **OTU190** | 663 | 0.46 | 526 | 0.31 | 4097 | 2.84 | 5286 | 1.16 | Acidimicrobiia | Acidimicrobiales | uncultured | uncultured |
| **OTU191** | 0 | 0.00 | 71 | 0.04 | 10 | 0.01 | 81 | 0.02 | Actinobacteria | Frankiales | Cryptosporangiaceae | Cryptosporangium |
| **OTU192** | 11 | 0.01 | 2790 | 1.65 | 18 | 0.01 | 2819 | 0.62 | Actinobacteria | Pseudonocardiales | Pseudonocardiaceae | uncultured |
| **OTU194** | 154 | 0.11 | 57 | 0.03 | 623 | 0.43 | 834 | 0.18 | Acidimicrobiia | Acidimicrobiales | uncultured | uncultured |
| **OTU195** | 0 | 0.00 | 22 | 0.01 | 0 | 0.00 | 22 | 0.00 | Actinobacteria | Micrococcales | Promicromonosporaceae | Promicromonospora |
| **OTU196** | 0 | 0.00 | 0 | 0.00 | 11 | 0.01 | 11 | 0.00 | Actinobacteria | Streptosporangiales | Streptosporangiaceae | unclassified |
| **OTU198** | 2 | 0.00 | 21 | 0.01 | 1 | 0.00 | 24 | 0.01 | Actinobacteria | Streptomycetales | Streptomycetaceae | Streptomyces |
| **OTU2** | 447 | 0.31 | 48903 | 28.89 | 3076 | 2.13 | 52426 | 11.47 | Actinobacteria | Pseudonocardiales | Pseudonocardiaceae | unclassified |
| **OTU20** | 1022 | 0.71 | 798 | 0.47 | 1176 | 0.81 | 2996 | 0.66 | Acidimicrobiia | Acidimicrobiales | Iamiaceae | uncultured |
| **OTU201** | 0 | 0.00 | 23 | 0.01 | 0 | 0.00 | 23 | 0.01 | Actinobacteria | Micrococcales | Micrococcaceae | Kocuria |
| **OTU202** | 0 | 0.00 | 0 | 0.00 | 8 | 0.01 | 8 | 0.00 | Acidimicrobiia | Acidimicrobiales | uncultured | uncultured |
| **OTU203** | 2483 | 1.73 | 528 | 0.31 | 8553 | 5.92 | 11564 | 2.53 | Acidimicrobiia | Acidimicrobiales | uncultured | uncultured |
| **OTU204** | 26 | 0.02 | 15 | 0.01 | 0 | 0.00 | 41 | 0.01 | Actinobacteria | Corynebacteriales | Corynebacteriaceae | uncultured |
| **OTU205** | 0 | 0.00 | 0 | 0.00 | 7 | 0.00 | 7 | 0.00 | Actinobacteria | Corynebacteriales | Corynebacteriaceae | Corynebacterium |
| **OTU206** | 0 | 0.00 | 0 | 0.00 | 11 | 0.01 | 11 | 0.00 | Actinobacteria | Propionibacteriales | Nocardioidaceae | Marmoricola |
| **OTU208** | 0 | 0.00 | 20 | 0.01 | 0 | 0.00 | 20 | 0.00 | Acidimicrobiia | Acidimicrobiales | uncultured | uncultured |
| **OTU21** | 1145 | 0.80 | 330 | 0.19 | 494 | 0.34 | 1969 | 0.43 | Actinobacteria | Streptomycetales | Streptomycetaceae | Streptomyces |
| **OTU210** | 3 | 0.00 | 22 | 0.01 | 61 | 0.04 | 86 | 0.02 | Actinobacteria | Micromonosporales | Micromonosporaceae | Luedemannella |
| **OTU212** | 0 | 0.00 | 0 | 0.00 | 3 | 0.00 | 3 | 0.00 | unclasiffied | unclassified | unclassified | unclassified |
| **OTU216** | 208 | 0.15 | 49 | 0.03 | 7 | 0.00 | 264 | 0.06 | Actinobacteria | Micromonosporales | Micromonosporaceae | uncultured |
| **OTU217** | 0 | 0.00 | 0 | 0.00 | 9 | 0.01 | 9 | 0.00 | unclasiffied | unclassified | unclassified | uncultured |
| **OTU219** | 3 | 0.00 | 9 | 0.01 | 18 | 0.01 | 30 | 0.01 | Thermoleophilia | Gaiellales | Gaiellaceae | uncultured |
| **OTU22** | 2300 | 1.61 | 271 | 0.16 | 88 | 0.06 | 2659 | 0.58 | Actinobacteria | Propionibacteriales | Propionibacteriaceae | unclassified |
| **OTU220** | 0 | 0.00 | 18 | 0.01 | 0 | 0.00 | 18 | 0.00 | Actinobacteria | Micrococcales | Intrasporangiaceae | unclassified |
| **OTU223** | 5 | 0.00 | 0 | 0.00 | 4 | 0.00 | 9 | 0.00 | Thermoleophilia | Gaiellales | uncultured | uncultured |
| **OTU224** | 0 | 0.00 | 17 | 0.01 | 0 | 0.00 | 17 | 0.00 | Actinobacteria | Propionibacteriales | Propionibacteriaceae | Tessaracoccus |
| **OTU225** | 0 | 0.00 | 0 | 0.00 | 10 | 0.01 | 10 | 0.00 | Actinobacteria | Pseudonocardiales | Pseudonocardiaceae | Pseudonocardia |
| **OTU227** | 0 | 0.00 | 0 | 0.00 | 7 | 0.00 | 7 | 0.00 | Actinobacteria | Streptomycetales | Streptomycetaceae | Streptomyces |
| **OTU229** | 2 | 0.00 | 0 | 0.00 | 7 | 0.00 | 9 | 0.00 | Actinobacteria | Micromonosporales | Micromonosporaceae | Catellatospora |
| **OTU23** | 386 | 0.27 | 2016 | 1.19 | 149 | 0.10 | 2551 | 0.56 | Actinobacteria | Micrococcales | Micrococcaceae | Arthrobacter |
| **OTU230** | 0 | 0.00 | 0 | 0.00 | 4 | 0.00 | 4 | 0.00 | Actinobacteria | Streptomycetales | Streptomycetaceae | Streptomyces |
| **OTU231** | 0 | 0.00 | 0 | 0.00 | 7 | 0.00 | 7 | 0.00 | Actinobacteria | Streptomycetales | Streptomycetaceae | Streptomyces |
| **OTU232** | 0 | 0.00 | 0 | 0.00 | 8 | 0.01 | 8 | 0.00 | Acidimicrobiia | Acidimicrobiales | uncultured | uncultured |
| **OTU233** | 49 | 0.03 | 14 | 0.01 | 1 | 0.00 | 64 | 0.01 | Actinobacteria | Micromonosporales | Micromonosporaceae | unclassified |
| **OTU234** | 0 | 0.00 | 18 | 0.01 | 42 | 0.03 | 60 | 0.01 | Actinobacteria | Frankiales | uncultured | uncultured |
| **OTU236** | 0 | 0.00 | 17 | 0.01 | 4 | 0.00 | 21 | 0.00 | Actinobacteria | Micromonosporales | Micromonosporaceae | unclassified |
| **OTU238** | 0 | 0.00 | 0 | 0.00 | 8 | 0.01 | 8 | 0.00 | Acidimicrobiia | Acidimicrobiales | uncultured | uncultured |
| **OTU239** | 3 | 0.00 | 122 | 0.07 | 30 | 0.02 | 155 | 0.03 | Actinobacteria | Pseudonocardiales | Pseudonocardiaceae | Pseudonocardia |
| **OTU24** | 2769 | 1.93 | 294 | 0.17 | 113 | 0.08 | 3176 | 0.70 | Actinobacteria | Streptosporangiales | Streptosporangiaceae | Planotetraspora |
| **OTU242** | 127 | 0.09 | 24 | 0.01 | 8 | 0.01 | 159 | 0.03 | Actinobacteria | Streptomycetales | Streptomycetaceae | Streptomyces |
| **OTU243** | 0 | 0.00 | 34 | 0.02 | 18 | 0.01 | 52 | 0.01 | Actinobacteria | Frankiales | Sporichthyaceae | Sporichthya |
| **OTU245** | 2 | 0.00 | 13 | 0.01 | 7 | 0.00 | 22 | 0.00 | Actinobacteria | Micrococcales | Micrococcaceae | Rothia |
| **OTU248** | 0 | 0.00 | 0 | 0.00 | 7 | 0.00 | 7 | 0.00 | Actinobacteria | Micromonosporales | Micromonosporaceae | uncultured |
| **OTU25** | 802 | 0.56 | 144 | 0.09 | 1203 | 0.83 | 2149 | 0.47 | Actinobacteria | Corynebacteriales | Nocardiaceae | Nocardia |
| **OTU251** | 1109 | 0.77 | 1100 | 0.65 | 1614 | 1.12 | 3823 | 0.84 | Actinobacteria | Pseudonocardiales | Pseudonocardiaceae | Pseudonocardia |
| **OTU252** | 0 | 0.00 | 0 | 0.00 | 3 | 0.00 | 3 | 0.00 | Actinobacteria | Frankiales | Frankiaceae | Frankia |
| **OTU253** | 0 | 0.00 | 10 | 0.01 | 0 | 0.00 | 10 | 0.00 | unclasiffied | unclassified | unclassified | unclassified |
| **OTU254** | 0 | 0.00 | 0 | 0.00 | 10 | 0.01 | 10 | 0.00 | Actinobacteria | Corynebacteriales | Mycobacteriaceae | Mycobacterium |
| **OTU256** | 0 | 0.00 | 0 | 0.00 | 3 | 0.00 | 3 | 0.00 | Actinobacteria | Propionibacteriales | Nocardioidaceae | Marmoricola |
| **OTU257** | 0 | 0.00 | 0 | 0.00 | 17 | 0.01 | 17 | 0.00 | Actinobacteria | Propionibacteriales | Nocardioidaceae | Nocardioides |
| **OTU258** | 283 | 0.20 | 96 | 0.06 | 320 | 0.22 | 699 | 0.15 | Acidimicrobiia | Acidimicrobiales | uncultured | uncultured |
| **OTU26** | 834 | 0.58 | 105 | 0.06 | 401 | 0.28 | 1340 | 0.29 | Actinobacteria | Streptomycetales | Streptomycetaceae | Streptomyces |
| **OTU261** | 0 | 0.00 | 0 | 0.00 | 3 | 0.00 | 3 | 0.00 | Actinobacteria | Propionibacteriales | Nocardioidaceae | Aeromicrobium |
| **OTU262** | 4942 | 3.45 | 6970 | 4.12 | 8508 | 5.89 | 20420 | 4.47 | Actinobacteria | Pseudonocardiales | Pseudonocardiaceae | Pseudonocardia |
| **OTU266** | 0 | 0.00 | 12 | 0.01 | 10 | 0.01 | 22 | 0.00 | Actinobacteria | Corynebacteriales | Nocardiaceae | Rhodococcus |
| **OTU268** | 0 | 0.00 | 9 | 0.01 | 0 | 0.00 | 9 | 0.00 | Actinobacteria | Corynebacteriales | Corynebacteriaceae | Corynebacterium |
| **OTU27** | 344 | 0.24 | 77 | 0.05 | 609 | 0.42 | 1030 | 0.23 | Acidimicrobiia | Acidimicrobiales | uncultured | uncultured |
| **OTU271** | 0 | 0.00 | 6 | 0.00 | 13 | 0.01 | 19 | 0.00 | Actinobacteria | Pseudonocardiales | Pseudonocardiaceae | Pseudonocardia |
| **OTU272** | 0 | 0.00 | 0 | 0.00 | 2 | 0.00 | 2 | 0.00 | Actinobacteria | Streptomycetales | Streptomycetaceae | Streptomyces |
| **OTU273** | 406 | 0.28 | 105 | 0.06 | 78 | 0.05 | 589 | 0.13 | Acidimicrobiia | Acidimicrobiales | uncultured | uncultured |
| **OTU274** | 0 | 0.00 | 0 | 0.00 | 3 | 0.00 | 3 | 0.00 | unclasiffied | unclassified | unclassified | uncultured |
| **OTU28** | 271 | 0.19 | 266 | 0.16 | 482 | 0.33 | 1019 | 0.22 | Acidimicrobiia | Acidimicrobiales | uncultured | uncultured |
| **OTU280** | 0 | 0.00 | 8 | 0.00 | 0 | 0.00 | 8 | 0.00 | Actinobacteria | Propionibacteriales | Propionibacteriaceae | Ponticoccus |
| **OTU281** | 0 | 0.00 | 0 | 0.00 | 4 | 0.00 | 4 | 0.00 | Actinobacteria | Propionibacteriales | Nocardioidaceae | Nocardioides |
| **OTU282** | 0 | 0.00 | 0 | 0.00 | 5 | 0.00 | 5 | 0.00 | Actinobacteria | Pseudonocardiales | Pseudonocardiaceae | Amycolatopsis |
| **OTU284** | 0 | 0.00 | 9 | 0.01 | 4 | 0.00 | 13 | 0.00 | Actinobacteria | Micrococcales | Micrococcaceae | Arthrobacter |
| **OTU289** | 0 | 0.00 | 0 | 0.00 | 1 | 0.00 | 1 | 0.00 | Actinobacteria | Propionibacteriales | Nocardioidaceae | Nocardioides |
| **OTU29** | 383 | 0.27 | 197 | 0.12 | 386 | 0.27 | 966 | 0.21 | Acidimicrobiia | Acidimicrobiales | Acidimicrobiaceae | uncultured |
| **OTU292** | 0 | 0.00 | 0 | 0.00 | 3 | 0.00 | 3 | 0.00 | Actinobacteria | Corynebacteriales | Corynebacteriaceae | Corynebacterium |
| **OTU294** | 0 | 0.00 | 0 | 0.00 | 2 | 0.00 | 2 | 0.00 | Actinobacteria | Propionibacteriales | Nocardioidaceae | Kribbella |
| **OTU296** | 46 | 0.03 | 0 | 0.00 | 73 | 0.05 | 119 | 0.03 | Acidimicrobiia | Acidimicrobiales | uncultured | uncultured |
| **OTU299** | 0 | 0.00 | 0 | 0.00 | 2 | 0.00 | 2 | 0.00 | Acidimicrobiia | Acidimicrobiales | uncultured | uncultured |
| **OTU3** | 750 | 0.52 | 13210 | 7.80 | 7032 | 4.87 | 20992 | 4.59 | Actinobacteria | Pseudonocardiales | Pseudonocardiaceae | Pseudonocardia |
| **OTU30** | 3025 | 2.11 | 214 | 0.13 | 454 | 0.31 | 3693 | 0.81 | Actinobacteria | Streptomycetales | Streptomycetaceae | Streptomyces |
| **OTU301** | 0 | 0.00 | 6 | 0.00 | 0 | 0.00 | 6 | 0.00 | Actinobacteria | unclasiffied | unclassified | unclassified |
| **OTU306** | 0 | 0.00 | 2 | 0.00 | 0 | 0.00 | 2 | 0.00 | Thermoleophilia | Gaiellales | uncultured | uncultured |
| **OTU309** | 3 | 0.00 | 1 | 0.00 | 16 | 0.01 | 20 | 0.00 | Acidimicrobiia | Acidimicrobiales | uncultured | uncultured |
| **OTU31** | 26 | 0.02 | 17 | 0.01 | 531 | 0.37 | 574 | 0.13 | unclasiffied | unclasiffied | unclassified | uncultured |
| **OTU316** | 0 | 0.00 | 0 | 0.00 | 4 | 0.00 | 4 | 0.00 | Actinobacteria | Propionibacteriales | Nocardioidaceae | Nocardioides |
| **OTU317** | 0 | 0.00 | 0 | 0.00 | 2 | 0.00 | 2 | 0.00 | Actinobacteria | Pseudonocardiales | Pseudonocardiaceae | Umezawaea |
| **OTU32** | 314 | 0.22 | 52 | 0.03 | 1324 | 0.92 | 1690 | 0.37 | Actinobacteria | unclasiffied | unclasiffied | unclassified |
| **OTU325** | 0 | 0.00 | 3 | 0.00 | 1 | 0.00 | 4 | 0.00 | Thermoleophilia | Gaiellales | uncultured | uncultured |
| **OTU330** | 0 | 0.00 | 27 | 0.02 | 145 | 0.10 | 172 | 0.04 | Actinobacteria | Micromonosporales | Micromonosporaceae | Rhizocola |
| **OTU34** | 67 | 0.05 | 96 | 0.06 | 477 | 0.33 | 640 | 0.14 | Acidimicrobiia | Acidimicrobiales | uncultured | uncultured |
| **OTU345** | 400 | 0.28 | 77 | 0.05 | 12 | 0.01 | 489 | 0.11 | Actinobacteria | Frankiales | Frankiaceae | Frankia |
| **OTU35** | 932 | 0.65 | 315 | 0.19 | 82 | 0.06 | 1329 | 0.29 | Actinobacteria | Micrococcales | Micrococcaceae | Arthrobacter |
| **OTU351** | 0 | 0.00 | 5 | 0.00 | 0 | 0.00 | 5 | 0.00 | Actinobacteria | Corynebacteriales | Corynebacteriaceae | Corynebacterium |
| **OTU36** | 312 | 0.22 | 68 | 0.04 | 1385 | 0.96 | 1765 | 0.39 | Acidimicrobiia | Acidimicrobiales | uncultured | uncultured |
| **OTU360** | 576 | 0.40 | 282 | 0.17 | 2432 | 1.68 | 3290 | 0.72 | Acidimicrobiia | Acidimicrobiales | uncultured | uncultured |
| **OTU368** | 0 | 0.00 | 114 | 0.07 | 57 | 0.04 | 171 | 0.04 | Actinobacteria | Pseudonocardiales | Pseudonocardiaceae | uncultured |
| **OTU377** | 45 | 0.03 | 0 | 0.00 | 27 | 0.02 | 72 | 0.02 | Actinobacteria | Frankiales | Sporichthyaceae | unclassified |
| **OTU378** | 517 | 0.36 | 435 | 0.26 | 445 | 0.31 | 1397 | 0.31 | Acidimicrobiia | Acidimicrobiales | uncultured | uncultured |
| **OTU38** | 50 | 0.03 | 136 | 0.08 | 715 | 0.49 | 901 | 0.20 | Actinobacteria | Geodermatophilales | Geodermatophilaceae | uncultured |
| **OTU389** | 0 | 0.00 | 0 | 0.00 | 1 | 0.00 | 1 | 0.00 | Actinobacteria | Propionibacteriales | Propionibacteriaceae | Propionimicrobium |
| **OTU39** | 0 | 0.00 | 12 | 0.01 | 388 | 0.27 | 400 | 0.09 | Actinobacteria | Streptomycetales | Streptomycetaceae | Streptomyces |
| **OTU390** | 0 | 0.00 | 0 | 0.00 | 1 | 0.00 | 1 | 0.00 | Actinobacteria | Catenulisporales | Actinospicaceae | Actinospica |
| **OTU391** | 0 | 0.00 | 0 | 0.00 | 1 | 0.00 | 1 | 0.00 | Thermoleophilia | Gaiellales | Gaiellaceae | uncultured |
| **OTU393** | 72 | 0.05 | 0 | 0.00 | 53 | 0.04 | 125 | 0.03 | Actinobacteria | Micromonosporales | Micromonosporaceae | Catellatospora |
| **OTU399** | 138 | 0.10 | 0 | 0.00 | 0 | 0.00 | 138 | 0.03 | Actinobacteria | Micrococcales | Microbacteriaceae | Pseudoclavibacter |
| **OTU4** | 4801 | 3.35 | 1921 | 1.13 | 16568 | 11.47 | 23290 | 5.10 | Acidimicrobiia | Acidimicrobiales | uncultured | uncultured |
| **OTU40** | 108 | 0.08 | 161 | 0.10 | 276 | 0.19 | 545 | 0.12 | Actinobacteria | Pseudonocardiales | Pseudonocardiaceae | uncultured |
| **OTU401** | 0 | 0.00 | 0 | 0.00 | 3 | 0.00 | 3 | 0.00 | Thermoleophilia | Gaiellales | Gaiellaceae | uncultured |
| **OTU403** | 3 | 0.00 | 0 | 0.00 | 0 | 0.00 | 3 | 0.00 | Actinobacteria | Corynebacteriales | Nocardiaceae | Rhodococcus |
| **OTU408** | 2 | 0.00 | 116 | 0.07 | 963 | 0.67 | 1081 | 0.24 | Acidimicrobiia | Acidimicrobiales | Iamiaceae | uncultured |
| **OTU41** | 667 | 0.47 | 0 | 0.00 | 111 | 0.08 | 778 | 0.17 | Actinobacteria | Frankiales | Frankiaceae | Frankia |
| **OTU419** | 0 | 0.00 | 0 | 0.00 | 2 | 0.00 | 2 | 0.00 | Thermoleophilia | Gaiellales | uncultured | uncultured |
| **OTU42** | 202 | 0.14 | 328 | 0.19 | 282 | 0.20 | 812 | 0.18 | Acidimicrobiia | Acidimicrobiales | uncultured | uncultured |
| **OTU421** | 128 | 0.09 | 234 | 0.14 | 128 | 0.09 | 490 | 0.11 | Actinobacteria | Corynebacteriales | Nocardiaceae | Rhodococcus |
| **OTU423** | 170 | 0.12 | 133 | 0.08 | 78 | 0.05 | 381 | 0.08 | Acidimicrobiia | Acidimicrobiales | uncultured | uncultured |
| **OTU424** | 0 | 0.00 | 0 | 0.00 | 120 | 0.08 | 120 | 0.03 | Acidimicrobiia | Acidimicrobiales | Acidimicrobiaceae | uncultured |
| **OTU425** | 0 | 0.00 | 0 | 0.00 | 1 | 0.00 | 1 | 0.00 | Thermoleophilia | Gaiellales | uncultured | uncultured |
| **OTU432** | 5272 | 3.68 | 2375 | 1.40 | 2392 | 1.66 | 10039 | 2.20 | Actinobacteria | Pseudonocardiales | Pseudonocardiaceae | Pseudonocardia |
| **OTU439** | 0 | 0.00 | 0 | 0.00 | 2 | 0.00 | 2 | 0.00 | Thermoleophilia | Gaiellales | uncultured | uncultured |
| **OTU44** | 1145 | 0.80 | 233 | 0.14 | 382 | 0.26 | 1760 | 0.39 | Acidimicrobiia | Acidimicrobiales | uncultured | uncultured |
| **OTU440** | 0 | 0.00 | 0 | 0.00 | 1 | 0.00 | 1 | 0.00 | Thermoleophilia | Gaiellales | uncultured | uncultured |
| **OTU443** | 846 | 0.59 | 347 | 0.21 | 227 | 0.16 | 1420 | 0.31 | Acidimicrobiia | Acidimicrobiales | uncultured | uncultured |
| **OTU444** | 0 | 0.00 | 2 | 0.00 | 0 | 0.00 | 2 | 0.00 | Actinobacteria | Micrococcales | Microbacteriaceae | unclassified |
| **OTU45** | 457 | 0.32 | 31 | 0.02 | 205 | 0.14 | 693 | 0.15 | Actinobacteria | Corynebacteriales | Nocardiaceae | unclassified |
| **OTU451** | 4 | 0.00 | 0 | 0.00 | 85 | 0.06 | 89 | 0.02 | Acidimicrobiia | Acidimicrobiales | uncultured | uncultured |
| **OTU46** | 552 | 0.39 | 190 | 0.11 | 215 | 0.15 | 957 | 0.21 | Actinobacteria | Micromonosporales | Micromonosporaceae | Luedemannella |
| **OTU47** | 1080 | 0.75 | 438 | 0.26 | 1410 | 0.98 | 2928 | 0.64 | Acidimicrobiia | Acidimicrobiales | uncultured | uncultured |
| **OTU48** | 500 | 0.35 | 189 | 0.11 | 82 | 0.06 | 771 | 0.17 | Acidimicrobiia | Acidimicrobiales | uncultured | uncultured |
| **OTU49** | 168 | 0.12 | 109 | 0.06 | 269 | 0.19 | 546 | 0.12 | Actinobacteria | Micrococcales | Microbacteriaceae | Agromyces |
| **OTU5** | 5051 | 3.53 | 2875 | 1.70 | 8477 | 5.87 | 16403 | 3.59 | Acidimicrobiia | Acidimicrobiales | uncultured | uncultured |
| **OTU50** | 1254 | 0.88 | 913 | 0.54 | 534 | 0.37 | 2701 | 0.59 | Actinobacteria | Pseudonocardiales | Pseudonocardiaceae | uncultured |
| **OTU51** | 218 | 0.15 | 82 | 0.05 | 167 | 0.12 | 467 | 0.10 | Acidimicrobiia | Acidimicrobiales | uncultured | uncultured |
| **OTU52** | 120 | 0.08 | 36 | 0.02 | 133 | 0.09 | 289 | 0.06 | Actinobacteria | Propionibacteriales | Nocardioidaceae | Kribbella |
| **OTU53** | 126 | 0.09 | 0 | 0.00 | 79 | 0.05 | 205 | 0.04 | Acidimicrobiia | Acidimicrobiales | uncultured | uncultured |
| **OTU54** | 179 | 0.13 | 1551 | 0.92 | 665 | 0.46 | 2395 | 0.52 | Actinobacteria | Pseudonocardiales | Pseudonocardiaceae | Pseudonocardia |
| **OTU56** | 0 | 0.00 | 11 | 0.01 | 162 | 0.11 | 173 | 0.04 | unclasiffied | unclassified | unclassified | uncultured |
| **OTU57** | 259 | 0.18 | 312 | 0.18 | 32 | 0.02 | 603 | 0.13 | unclasiffied | unclassified | unclassified | uncultured |
| **OTU59** | 206 | 0.14 | 0 | 0.00 | 29 | 0.02 | 235 | 0.05 | unclasiffied | unclassified | unclassified | uncultured |
| **OTU6** | 10772 | 7.53 | 1692 | 1.00 | 5753 | 3.98 | 18217 | 3.99 | Acidimicrobiia | Acidimicrobiales | uncultured | uncultured |
| **OTU60** | 0 | 0.00 | 0 | 0.00 | 111 | 0.08 | 111 | 0.02 | Actinobacteria | Pseudonocardiales | Pseudonocardiaceae | Lentzea |
| **OTU61** | 440 | 0.31 | 75 | 0.04 | 122 | 0.08 | 637 | 0.14 | Actinobacteria | Streptomycetales | Streptomycetaceae | Streptomyces |
| **OTU62** | 416 | 0.29 | 114 | 0.07 | 101 | 0.07 | 631 | 0.14 | Actinobacteria | Nakamurellales | Nakamurellaceae | Nakamurella |
| **OTU63** | 62 | 0.04 | 264 | 0.16 | 142 | 0.10 | 468 | 0.10 | Acidimicrobiia | Acidimicrobiales | Acidimicrobiaceae | uncultured |
| **OTU64** | 493 | 0.34 | 30 | 0.02 | 78 | 0.05 | 601 | 0.13 | Actinobacteria | Micromonosporales | Micromonosporaceae | Actinoplanes |
| **OTU65** | 0 | 0.00 | 52 | 0.03 | 198 | 0.14 | 250 | 0.05 | Actinobacteria | Geodermatophilales | Geodermatophilaceae | Geodermatophilus |
| **OTU66** | 64 | 0.04 | 177 | 0.10 | 5 | 0.00 | 246 | 0.05 | Acidimicrobiia | Acidimicrobiales | Acidimicrobiaceae | uncultured |
| **OTU67** | 397 | 0.28 | 65 | 0.04 | 176 | 0.12 | 638 | 0.14 | Actinobacteria | Corynebacteriales | Mycobacteriaceae | Mycobacterium |
| **OTU68** | 98 | 0.07 | 55 | 0.03 | 33 | 0.02 | 186 | 0.04 | Actinobacteria | Micromonosporales | Micromonosporaceae | Actinoplanes |
| **OTU69** | 0 | 0.00 | 35 | 0.02 | 272 | 0.19 | 307 | 0.07 | Actinobacteria | Propionibacteriales | Propionibacteriaceae | unclassified |
| **OTU7** | 0 | 0.00 | 2875 | 1.70 | 4776 | 3.31 | 7651 | 1.67 | Actinobacteria | Pseudonocardiales | Pseudonocardiaceae | uncultured |
| **OTU72** | 221 | 0.15 | 13 | 0.01 | 9 | 0.01 | 243 | 0.05 | Actinobacteria | Corynebacteriales | Nocardiaceae | Nocardia |
| **OTU73** | 200 | 0.14 | 4 | 0.00 | 41 | 0.03 | 245 | 0.05 | Actinobacteria | Propionibacteriales | Nocardioidaceae | Nocardioides |
| **OTU75** | 111 | 0.08 | 30 | 0.02 | 98 | 0.07 | 239 | 0.05 | Actinobacteria | Frankiales | Cryptosporangiaceae | uncultured |
| **OTU76** | 37 | 0.03 | 70 | 0.04 | 27 | 0.02 | 134 | 0.03 | Actinobacteria | Micrococcales | Microbacteriaceae | unclassified |
| **OTU77** | 96 | 0.07 | 24 | 0.01 | 65 | 0.04 | 185 | 0.04 | Actinobacteria | Micrococcales | Microbacteriaceae | unclassified |
| **OTU78** | 151 | 0.11 | 18 | 0.01 | 0 | 0.00 | 169 | 0.04 | Actinobacteria | Corynebacteriales | Corynebacteriaceae | Corynebacterium |
| **OTU79** | 73 | 0.05 | 0 | 0.00 | 50 | 0.03 | 123 | 0.03 | Actinobacteria | unclassified | uncultured | uncultured |
| **OTU8** | 1075 | 0.75 | 23634 | 13.96 | 839 | 0.58 | 25548 | 5.59 | Actinobacteria | Pseudonocardiales | Pseudonocardiaceae | uncultured |
| **OTU80** | 86 | 0.06 | 21 | 0.01 | 202 | 0.14 | 309 | 0.07 | Acidimicrobiia | Acidimicrobiales | uncultured | uncultured |
| **OTU81** | 78 | 0.05 | 18 | 0.01 | 176 | 0.12 | 272 | 0.06 | Actinobacteria | Geodermatophilales | Geodermatophilaceae | Geodermatophilus |
| **OTU82** | 0 | 0.00 | 6 | 0.00 | 91 | 0.06 | 97 | 0.02 | Acidimicrobiia | Acidimicrobiales | Acidimicrobiaceae | uncultured |
| **OTU83** | 35 | 0.02 | 0 | 0.00 | 45 | 0.03 | 80 | 0.02 | Actinobacteria | Glycomycetales | Glycomycetaceae | Glycomyces |
| **OTU88** | 371 | 0.26 | 374 | 0.22 | 110 | 0.08 | 855 | 0.19 | Actinobacteria | Micromonosporales | Micromonosporaceae | Longispora |
| **OTU9** | 349 | 0.24 | 262 | 0.15 | 3451 | 2.39 | 4062 | 0.89 | Acidimicrobiia | Acidimicrobiales | uncultured | uncultured |
| **OTU91** | 108 | 0.08 | 48 | 0.03 | 5 | 0.00 | 161 | 0.04 | Actinobacteria | Micrococcales | Microbacteriaceae | Microbacterium |
| **OTU92** | 129 | 0.09 | 14 | 0.01 | 13 | 0.01 | 156 | 0.03 | Actinobacteria | Corynebacteriales | unclassified | unclassified |
| **OTU94** | 43 | 0.03 | 0 | 0.00 | 34 | 0.02 | 77 | 0.02 | Actinobacteria | Corynebacteriales | Nocardiaceae | Smaragdicoccus |
| **OTU95** | 53 | 0.04 | 0 | 0.00 | 26 | 0.02 | 79 | 0.02 | Actinobacteria | Micrococcales | Micrococcaceae | Arthrobacter |
| **OTU97** | 902 | 0.63 | 62 | 0.04 | 254 | 0.18 | 1218 | 0.27 | Actinobacteria | Corynebacteriales | Mycobacteriaceae | Mycobacterium |
| **OTU98** | 1414 | 0.99 | 1807 | 1.07 | 10021 | 6.94 | 13242 | 2.90 | Acidimicrobiia | Acidimicrobiales | uncultured | uncultured |
| **OTU99** | 1508 | 1.05 | 154 | 0.09 | 251 | 0.17 | 1913 | 0.42 | Actinobacteria | Streptomycetales | Streptomycetaceae | Streptomyces |
|  | 143130 |  | 169258 |  | 144490 |  | 456878 |  |  |  |  |  |
