# Supplementary material for: Isolation, Characterization, and Antibacterial Activity of Hard-to-Culture Actinobacteria from Cave Moonmilk Deposits
Source: Antibiotics (Basel). 2018 Mar 22;7(2):28. doi: 10.3390/antibiotics7020028 (PMC6023089; doi:10.3390/antibiotics7020028)
Supplement: Supplementary file 1 [file antibiotics-07-00028-s001.zip › Supplementary Table S2.docx]

**Supplementary Table S2**

**Accession number of 16S rRNA sequences of MMun strains.**

| **MMun strains** | **Accession numbers** | **MMun strains** | **Accession numbers** |
| --- | --- | --- | --- |
| MMun129 | MG980169 | MMun152 | MG980075 |
| MMun130 | MG980170 | MMun153 | MG980076 |
| MMun131 | MG980171 | MMun154 | MG980189 |
| MMun133 | MG980172 | MMun155 | MG980077 |
| MMun135 | MG980173 | MMun156 | MG980078 |
| MMun136 | MG980174 | MMun157 | MG980190 |
| MMun137 | MG980175 | MMun158 | MG980079 |
| MMun138 | MG980176 | MMun159 | MG980080 |
| MMun139 | MG980177 | MMun160 | MG980081 |
| MMun140 | MG980178 | MMun161 | MG980082 |
| MMun141 | MG980179 | MMun162 | MG980083 |
| MMun142 | MG980180 | MMun163 | MG980084 |
| MMun143 | MG980181 | MMun164 | MG980085 |
| MMun144 | MG980182 | MMun166 | MG980086 |
| MMun145 | MG980183 | MMun167 | MG980087 |
| MMun146 | MG980184 | MMun168 | MG980088 |
| MMun147 | MG980185 | MMun170 | MG980089 |
| MMun148 | MG980186 | MMun171 | MG980090 |
| MMun149 | MG980074 | MMun172 | MG980091 |
| MMun150 | MG980187 | MMun174 | MG980092 |
| MMun151 | MG980188 | MMun176 | MG980093 |
