# Supplementary figures and images for: Isolation, Characterization, and Antibacterial Activity of Hard-to-Culture Actinobacteria from Cave Moonmilk Deposits
Source: Antibiotics (Basel). 2018 Mar 22;7(2):28. doi: 10.3390/antibiotics7020028 (PMC6023089; doi:10.3390/antibiotics7020028)

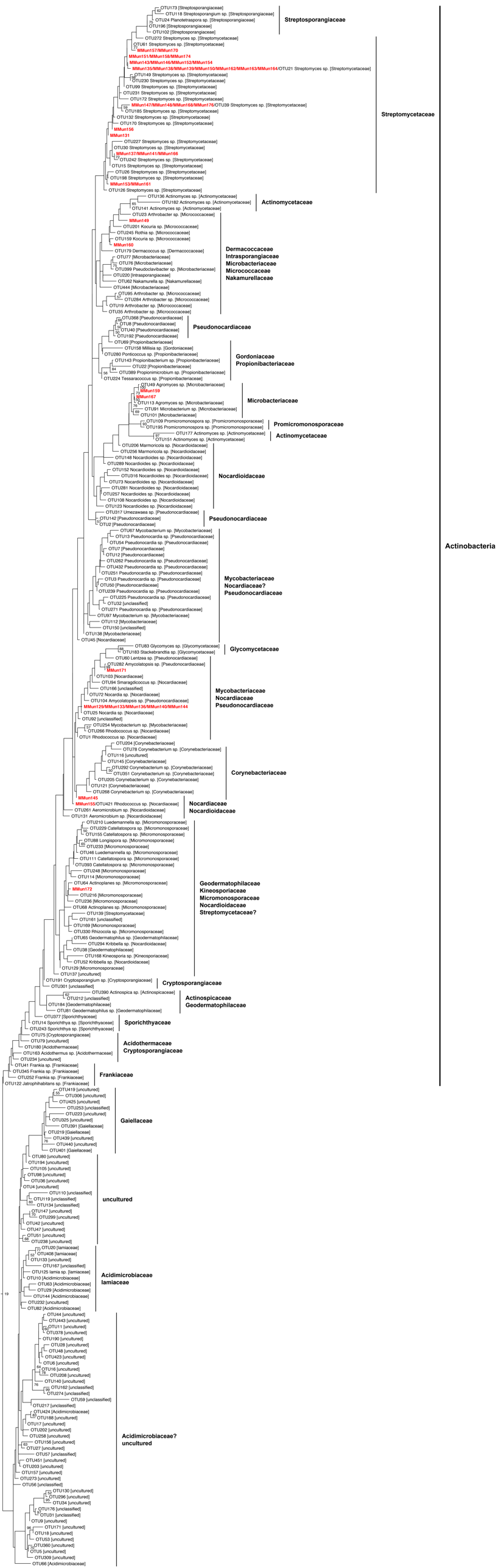

Supplement: Supplementary file 1 [file antibiotics-07-00028-s001.zip › Supplementary Figure S1.pdf]

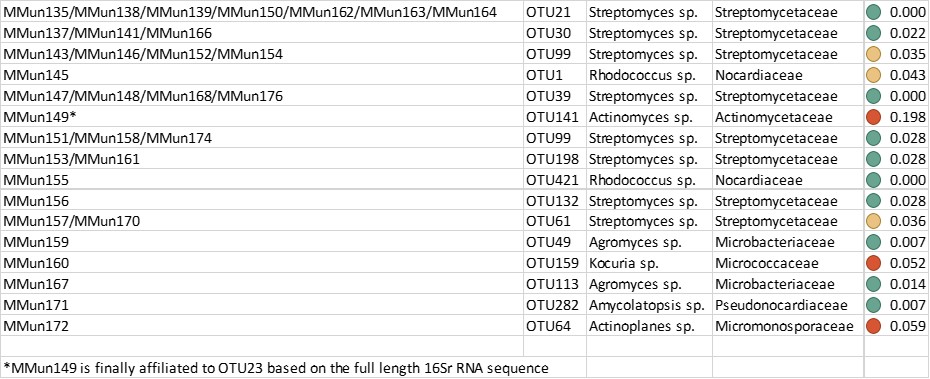

Supplement: Supplementary file 1 [file antibiotics-07-00028-s001.zip › Supplementary Figure S2.jpg]
